# Classical and alternative complement activation on photoreceptor outer segments drives monocyte-dependent retinal atrophy

**DOI:** 10.1038/s41598-018-25557-8

**Published:** 2018-05-09

**Authors:** Kenneth J. Katschke, Hongkang Xi, Christian Cox, Tom Truong, Yann Malato, Wyne P. Lee, Brent McKenzie, Rommel Arceo, Jianhua Tao, Linda Rangell, Mike Reichelt, Lauri Diehl, Justin Elstrott, Robby M Weimer, Menno van Lookeren Campagne

**Affiliations:** 1 Department of Immunology, Genentech, Inc, South San Francisco, CA 94080 USA; 2 Department of Translational Immunology, Genentech, Inc, South San Francisco, CA 94080 USA; 3 Department of Pathology, Genentech, Inc, South San Francisco, CA 94080 USA; 4 Department of Biomedical Imaging, Genentech, Inc, South San Francisco, CA 94080 USA

## Abstract

Geographic atrophy (GA), the advanced form of dry age-related macular degeneration (AMD), is characterized by progressive loss of retinal pigment epithelium cells and photoreceptors in the setting of characteristic extracellular deposits and remains a serious unmet medical need. While genetic predisposition to AMD is dominated by polymorphisms in complement genes, it remains unclear how complement activation contributes to retinal atrophy. Here we demonstrate that complement is activated on photoreceptor outer segments (POS) in the retina peripheral to atrophic lesions associated with GA. When exposed to human serum following outer blood-retinal barrier breakdown, POS act as potent activators of the classical and alternative complement pathway. In mouse models of retinal degeneration, classical and alternative pathway complement activation on photoreceptors contributed to the loss of photoreceptor function. This was dependent on C5a-mediated recruitment of peripheral blood monocytes but independent of resident microglia. Genetic or pharmacologic inhibition of both classical and alternative complement C3 and C5 convertases was required to reduce progressive degeneration of photoreceptor rods and cones. Our study implicates systemic classical and alternative complement proteins and peripheral blood monocytes as critical effectors of localized retinal degeneration with potential relevance for the contribution of complement activation to GA.

## Introduction

Age-related macular degeneration (AMD) is a leading cause of blindness in industrialized countries affecting approximately 3% of people over the age of 75. As the proportion of the elderly population increases, it will become an even larger medical problem that is predicted to affect 196 million people worldwide by 2020^1–3^. There are two main types of AMD: exudative, or neovascular AMD, which is characterized by disruption of the Bruch’s membrane and retinal pigment epithelium (RPE) with subsequent invasion of the retina by choroidal neovessels; and geographic atrophy (GA), which is associated with characteristic extracellular deposits and loss of RPE and photoreceptors^4,5^. While GA is generally seen as an avascular disease, quiescent microvasculature associated with GA has been observed in a subpopulation of patients^6,7^. Vision loss associated with neovascular AMD can be treated with VEGF inhibitors with favorable outcomes, but there are currently no approved or effective therapies for the treatment of GA^8^.

Genome-wide association studies have pointed to an important role for complement activation in AMD^9^. Complement activation on cell surfaces is initiated through three pathways: the classical pathway (CP), mannose-binding lectin (MBL) and alternative pathway (AP). Each pathway results in the formation of the C3 and C5 convertases, which are multi-subunit serine protease complexes critical for activation of the central complement proteins C3 and C5^10^. In turn, complement activation is kept in check through negative regulators present as membrane-anchored proteins on the cell surface (decay accelerating factor (CD55), membrane cofactor protein (CD46) and the membrane attack complex (MAC) inhibitor (CD59)) and as soluble proteins, including complement factor H (CFH) and complement factor I (CFI). AMD risk is increased in carriers of genetic variants that affect different aspects of the complement system, including negative regulation of complement activation (CFH and CFI), convertase formation (complement factor B (CFB), C2 and C3) and MAC (C5b-9) formation^11–16^. Studies in preclinical models of retinal degeneration have implicated complement activation in retinal cell loss^17–21^, but clinical studies using complement inhibitors to treat GA have shown mixed results^22,23^. Complement proteins have been found associated with drusen, extracellular deposits between the RPE basal lamina and the inner collagenous layer of the Bruch’s membrane that precede retinal degeneration^24^. How complement activation is associated with GA lesion growth or predisposes to earlier stages of the disease is unclear. Hence, further studies are necessary to determine the cellular and molecular basis for the strong genetic association of complement activation with AMD risk.

Photoreceptors are comprised of cell bodies in the outer nuclear layer (ONL) and the photoreceptor inner and photoreceptor outer segments (POS). POS expose phosphatidylserine (PS) on their surface, which is critical for their phagocytosis by RPE cells^25^. In GA, a lesion manifests as an area with photoreceptor and RPE cell loss that expands in the macula and leads to visual impairment in the form of scotomas (blind spots in the visual field) and progressively to a severe and irreversible decline in visual acuity once there is foveal involvement^26^. The lesion border is best characterized by the descent of the external limiting membrane (ELM) towards the Bruch’s membrane^27,^^28^. Approaching this ELM descent from the peripheral retina, the percentage of RPE cells with abnormal morphology increases and POS and photoreceptor numbers decrease^28,29^. Abnormal RPE and photoreceptor morphology in a junctional or transition zone ~500 μm peripheral of the lesion border corresponds to the loss of visual sensitivity as measured by micro-perimetry^30^. Subretinal mononuclear phagocytes (MPs^31^) are present in the transition zone and associated with cone outer segment and photoreceptor loss, pointing to a potential pathogenic role of MPs in the retina peripheral to the GA lesion^32,33^. The events that initiate MP recruitment to the transition zone have so far not been defined.

The current study demonstrates that complement activation in the outer retina leads to recruitment of monocytes and phagocytosis of complement C3-opsonized POS. Inhibiting all complement pathways upstream in the complement cascade was required for maximal protection of the retina. These new mechanistic insights could help us understand how complement activation contributes to retinal degeneration at the cellular and molecular level.

## Results

### Complement activation on photoreceptor outer segments (POS)

**Figure 1:**
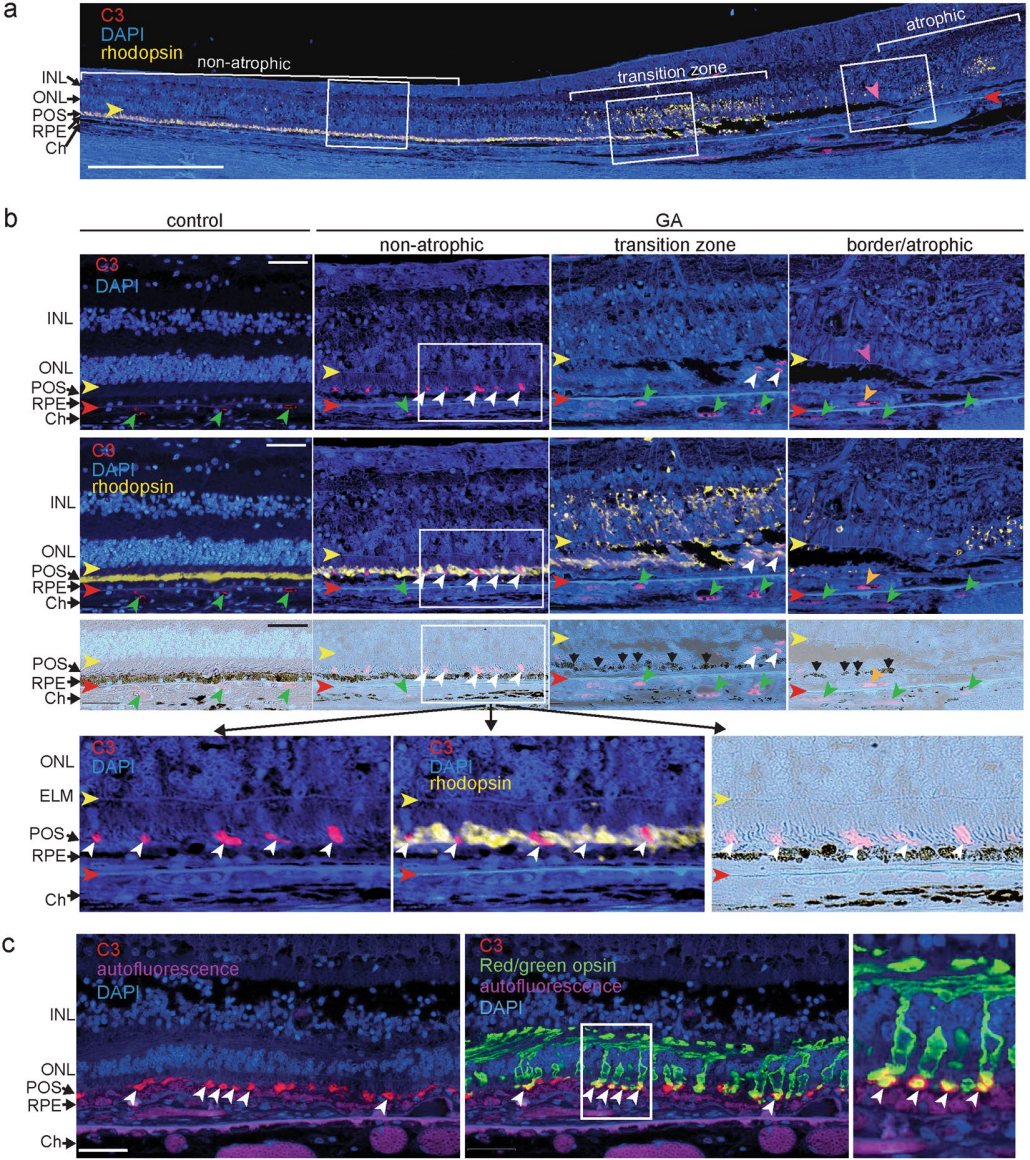
Complement C3 immunoreactivity on photoreceptor outer segments. (**a**) Cross-section through the retina of an 82 year old female donor diagnosed with GA 2 years prior to death. The section captures a non-atrophic area, a transition zone of photoreceptor degeneration recognized by mislocalized rhodopsin (yellow fluorescence) and the atrophic area that lacks photoreceptors. The GA lesion border (purple arrowhead) is noted by the decent of the external limiting membrane (ELM) towards the Bruch’s membrane (red arrowhead) and follows the GA transition zone where C3 immunoreactivity (red fluorescence) on POS was detected (boxed areas). Mislocalized rhodopsin in the atrophic zone likely represent degenerate rods. (**b**) Localization of C3 (red) and rhodopsin (yellow) immunoreactivity in a control donor eye (left panels) and a donor eye diagnosed with GA with close-ups of boxed insets of non-atrophic, transition and border/atrophic zones as displayed in (**a**). Top panels show C3 staining (red), the second row of panels show rhodopsin staining merged with C3 staining and the third row of panels are brightfield images indicating discontinuous RPE cells (black arrows). Bottom panels are close-ups of boxed inset of the non-atrophic zone showing C3 and rhodopsin staining in the POS layer. White arrowheads indicate C3 positive POS, green arrowheads indicate C3 staining in the choriocapillaris, orange arrowhead indicates sub-RPE neovasculature (right panel), yellow arrowheads indicate the external limiting membrane (ELM) and red arrowheads indicate the position of the Bruch’s membrane. (**c**) Dual immunofluorescence of complement C3 (red) and red/green cone opsin (green) in the eye of an 86 year old female donor diagnosed with GA. The image is taken ~1.2 mm from the edge of the GA lesion. Autofluorescence of RPE cells, Bruch’s membrane and choroidal vascular lumen is shown in the CFP channel (purple). White arrowheads indicate C3 immunoreactivity on cone POS. Scale bar = 0.5 mm (**a**) and 50 μm (**b**,**c**). INL = inner nuclear layer; ONL = outer nuclear layer; ELM = external limiting membrane; POS = photoreceptor outer segments; RPE = retinal pigmented epithelium; Ch = choroid.

Upon proteolytic activation, the complement proteins C3, required for all complement pathways, and complement C4, required for the CP and MBL pathway, covalently bind to cell surfaces that lack negative regulators of complement activation in a process termed “opsonization”. In order to determine whether specific cells or structures in the human retina are opsonized with C3 or C4, we performed immunohistochemistry on post-mortem eyes of control donors and donors diagnosed with AMD (Table 1). The AMD donor eyes selected for this study showed evidence of GA based on the donors’ ophthalmic history and on histopathological grading on sections^28,29^. The GA atrophic zone was defined by the lack of photoreceptors and a continuous layer of RPE cells (Fig. 1a,b, Supplemental Fig. 1a)^26,29,34^. The GA border was defined by the descent of the ELM towards the Bruch’s membrane^28^. The region immediately adjacent to the GA border, the transition zone, showed rhodopsin mislocalization from the outer segments to photoreceptor cell bodies as the outer segments degenerate (Fig. 1a,b).

**Table 1:** Donor characteristics and C3/C4 staining on POS.

	Mean age (±SD)	Donors	Females/males	Total Eyes	C3^+^ POS	C4^+^ POS
AMD	85.9 (±5.1)	13	9/4	19	5/19	12/18
Control	76.4 (±8.4)	9	4/5	13	0/11	1/13

C3 and C4 were detected in the vascular lumen of choroid and choriocapillaris of all human control and AMD donors (Fig. 1b and Supplemental Fig. 1b). Complement C3 and C4 immunoreactivity was found in drusen of the peripheral retina in <50% of donor eyes (control and AMD) consistent with our previous observations^35^. In eyes of donors diagnosed with GA, C3 and C4 immunostaining was occasionally present in the outer retinal layers 500–1000 μm peripheral to the GA lesion border, an area where noticeable pathology has been observed^28^. In a subset of donor eyes (Table 1), C3 and C4 immunoreactivity was present on POS located approximately 1.5 mm peripheral to the superior and inferior border of the atrophic zone and approximately 3.0 mm superior and inferior of the fovea (Fig. 1a,b, and Table 2). Using co-localization with a cone-specific opsin, we demonstrate that complement C3 immunoreactivity was found on a subset of cone outer segments and not on the cone cell body of AMD donor eyes (Fig. 1c). How serum complement proteins access photoreceptor outer segments in the undetached retina distal from the lesion border remains unclear. Complement C3 and C4 were absent in 12 out of 13 control donor eyes (Table 1). Presence of C4 immunoreactivity in one of the control donor eyes may be explained by the presence of early stage disease in this eye that was not diagnosed prior to death or visible in cross-sections through the macula. In a separate series of donor eyes, we determined if other complement proteins are found in the POS layer. CFB, which is a serum protein required for alternative pathway complement activation, was found between the photoreceptor cell bodies and the Bruch’s membrane in donor eyes diagnosed with AMD, but not in control donor eyes (Supplemental Fig. 2). The absence of selective localization of CFB to POS may be due to the fact that it is not an opsonin but a serum protein that may distribute to the photoreceptor inner segments. Antibody specificity was confirmed using isotope-matched antibodies on consecutive sections and pellets of cells incubated with complement-sufficient or -deficient sera (Supplemental Figs 2, 3 and^35^). In sum, apart from the vascular lumen, C3 and C4 immunoreactivity was below detection in the neural retina of control donor eyes and below detection in the atrophic area. In a subset of photoreceptors and a subset of donor eyes diagnosed with GA, C3 and C4 staining was restricted to POS of both rods and cones at a location distal from the GA lesion. This indicates that complement may be implicated in cellular processes at early stages of the disease.

**Table 2:** GA concentricity.

	Length of atrophic zone (mm)			Location of complement immunoreactivity (mm)			
	Superior	Inferior	Sum	From Fovea		From edge of GA	
				Superior	Inferior	Superior	Inferior
Avg ± SD (n)	2.2 ± 1.5 (11)	1.5 ± 0.9 (10)	3.5 ± 2.4 (11)	2.6 ± 2.1 (5)	3.5 ± 4.0 (7)	1.3 ± 1.6 (5)	2.0 ± 3.7 (8)

**Figure 2:**
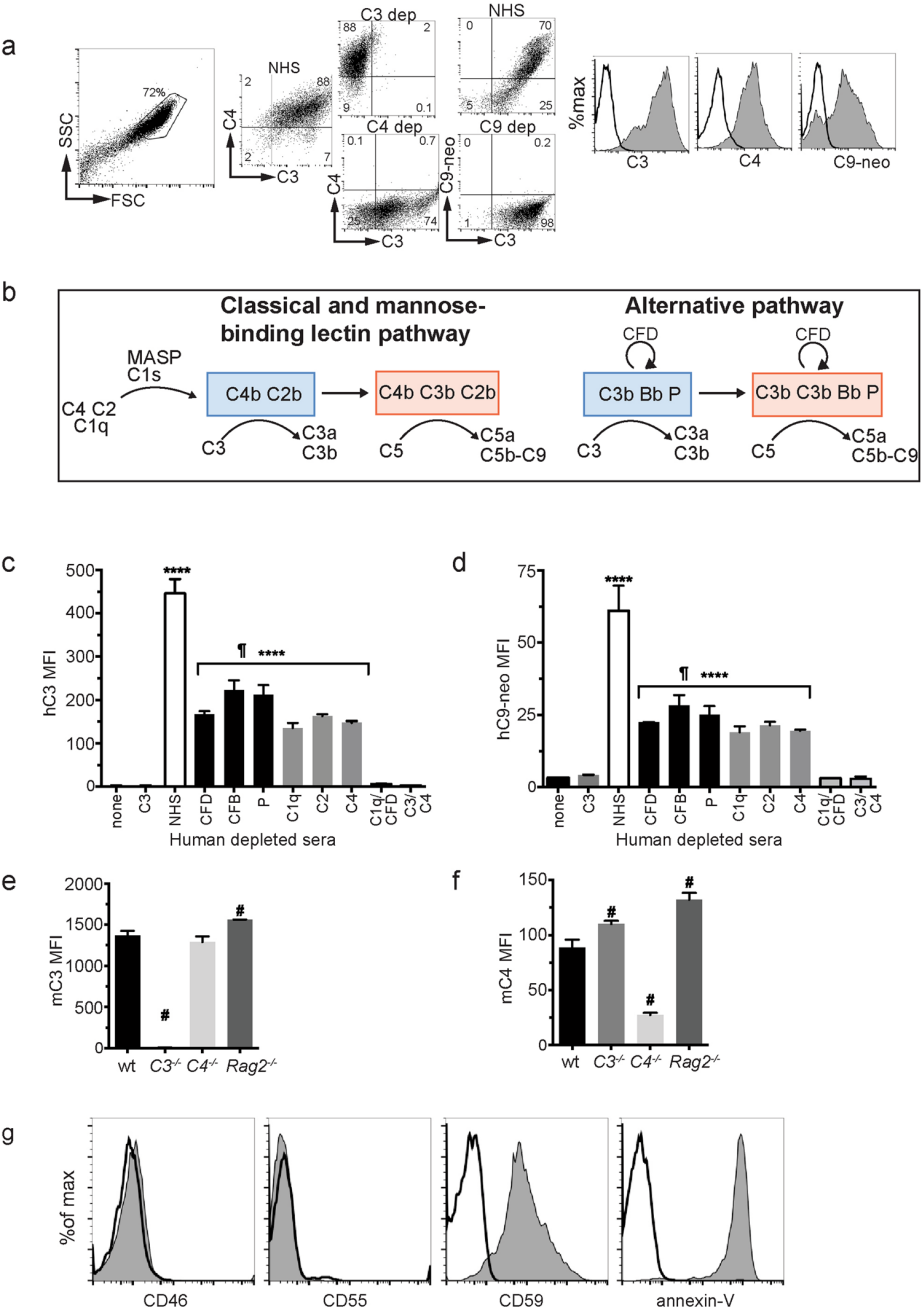
Photoreceptor outer segments activate both the classical and alternative complement pathway. (**a**) Flow cytometry of C3, C4 and C9 neoepitope proteins on the surface of bovine POS following incubation with normal human serum (NHS, shaded area in histograms) vs C3, C4, C9 depleted serum (solid lines in histograms). (**b**) Diagram of molecular components of the AP and CP/MBL complement pathways. C3 and C5 convertases are indicated in blue and red boxes, respectively. (**c**,**d**) Bovine POS were incubated with human sera depleted of various complement proteins and quantified by flow cytometry using a C3-specific antibody (**c**) or a C9 neo-epitope-specific antibody (**d**), n = 3. (**e** and **f**) Bovine POS were incubated with mouse wt, C3 deficient, C4 deficient, or Ig deficient (*Rag2*^*−/−*^) serum, surface stained for C3 (**e**) or C4 (**f**) protein and quantified by flow cytometry. (**g**) Flow cytometry analysis of cell-surface complement regulatory proteins and annexin V binding to bovine POS, solid line is isotype control and shaded area is indicated antibody. ^¶^p < 0.0001 NHS vs all other sera, ****p < 0.0001 vs C1q/CFD depleted serum, ^#^p < 0.001 vs mouse wt serum by 1-way ANOVA with Dunnet’s multiple comparisons test. Error bars are SD for triplicate repeats, the experiments were repeated three times with similar results. NHS = normal human serum, CFD = complement factor D, CFB = complement factor B, P = properdin, MFI = mean fluorescence intensity.

To determine which complement pathways are required for C3, C4 and C5 activation on POS, we established an *in vitro* system using isolated bovine POS and human sera in which various complement proteins were selectively depleted. POS incubated with 20% normal human serum activated C3, C4 and C5 on their surface as demonstrated by flow cytometry (Fig. 2a). Both classical (C1q, C2, C4-dependent) and alternative (properdin (P), complement factor D (CFD)- and CFB-dependent) pathways contributed to complement C3 and C5b-9 (MAC) activation on POS (Fig. 2b–d). Combined depletion of C1q and CFD fully suppressed complement activation to levels found in the absence of serum, indicating that the MBL pathway did not contribute to complement activation on POS *in vitro*^10^. Similarly, bovine POS incubated with mouse serum activated C3 and C4 on their surface and this process was independent of serum Ig’s as serum from RAG2^−/−^ mice that lack Ig-producing B-cells, was able to opsonize POS with C3 and C4 (Fig. 2e,f). Consistent with a low threshold for complement activation on the POS, bovine POS lacked the complement regulatory proteins CD46 and CD55, but expressed the MAC-inhibitory protein CD59 (Fig. 2g). Annexin V bound to the surface of POS, confirming PS exposure^25,36^. C1q can bind to surface-exposed PS directly^37^ or indirectly via Annexin V^38^ to trigger classical pathway complement activation, consistent with the lack of Ig requirement for opsonization. Mouse photoreceptors that lack cell surface regulators of complement activation opsonized with C3 and C4 when incubated with complement sufficient, but not complement deficient, mouse serum (Supplemental Fig. 4). In contrast, CD3^+^ lymphocytes which express the complement regulatory proteins CD46 and CD55, showed minimal C3 and C4 opsonization when exposed to mouse serum, compatible with high expression of the membrane-bound complement convertase inhibitors CD46 and CD55.

### Complement activates on POS following blood-retina barrier disruption in mice

**Figure 3:**
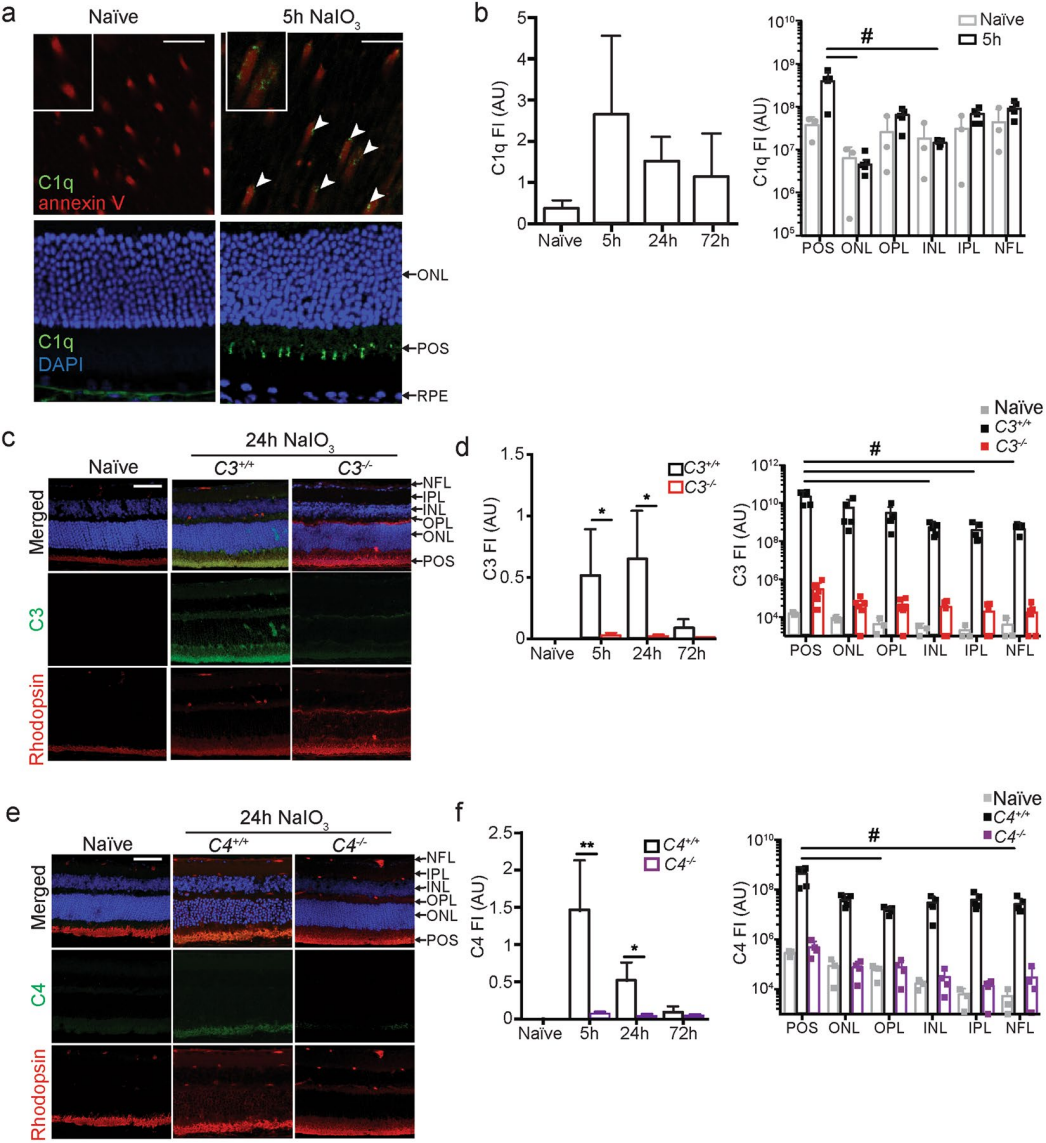
Complement activates on mouse photoreceptor outer segments following RPE cell loss. (**a**) Co-localization of PS (red, detected by annexin V) and C1q (green, white arrows) on murine POS 5 h following NaIO_3_ administration. Top panel is a retina flatmount (boxed inset is a close-up of a subset of POS) and bottom panel is a coronal section through the retina. (**b**) C1q staining was quantified at 5, 24 and 72 hours post NaIO_3_. The right graph quantifies C1q staining in the retina layers 5 h after NaIO_3_ administration. (**c**) Complement C3 (green) immunostaining in the retina of a naïve and a *C3*^+/+^ and *C3*^−/−^ mouse 24 h after NaIO_3_ administration. (**d**) Left graphs show quantification of C3 at 5, 24, and 72 hours after NaIO_3_. Right graph quantifies complement immunoreactivity at 24 h in each retinal layer, n = 5. (**e**) Complement C4 (green) immunoreactivity in the retina of a naïve and a *C4*^+/+^ and *C4*^−/−^ mouse 24 h after NaIO_3_ administration. (**f**) Left graphs show quantification of C4 at 5, 24, and 72 hours after NaIO_3_. Right graph quantifies complement at 24 h in each retina layer, n = 5. Scale bars = 10 μm. Naïve = not NaIO_3_ treated. FI = Fluorescent Intensity expressed as arbitrary units (AU). *C3*^−/−^ and *C4*^−/−^ mice were included as negative controls for the immunoreactivity. Experiments were repeated 2–3 times with similar results. Error bars are SDs. *p < 0.05 **p < 0.01 by Mann-Whitney two-tailed test; ^#^p < 0.05 by 1-way ANOVA Kruskal-Wallis with Dunn’s multiple comparisons test. POS = photoreceptor outer segments; ONL = outer nuclear layer; OPL = outer plexiform layer; INL = inner nuclear layer; IPL = inner plexiform layer; NFL = nerve fiber layer.

In the donor eyes that showed C3 immunoreactivity on POS, we identified sub-foveal neo-vessels that stained positive for PLVAP, a marker for fenestrated capillaries of the choroid (Supplemental Fig. 5). Choroidal neovascularization has been observed in a sub-population of patients diagnosed with GA and points to disruption of the outer blood-retinal barrier^6,7^. To address the *in vivo* consequences of complement activation on POS, we recapitulated RPE cell death and loss of outer blood-retina barrier (BRB) function by treating mice with NaIO_3_^39^ that induces death of RPE within hours^40,41^. This is accompanied by transient edema as measured by spectral domain-optical coherence imaging (SD-OCT)^42^ and fluorescein dye and albumin leakage^39^. NaIO_3_ acutely kills RPE cells and the model therefore does not recapitulate several pathophysiological aspects of GA, including the formation of drusen and the formation of dense sub-RPE deposits that precede progressive RPE cell death. The model may recapitulate some of the events leading to presentation of GA, in which RPE cells fail, POS are lost and the photoreceptors die^34,43^, accompanied by an increased presence of MP in the choroid as well as in the outer retina layers^44–46^. Complement activation on POS *in vivo* was determined by immunohistochemistry at various time points following NaIO_3_ administration. PS exposure on POS allows for their recognition by phagocytosing RPE cells and is critical for proper maintenance of the visual cycle^25^. PS can also bind the globular domain of C1q directly^37^ or indirectly via annexin A2 and A5^38^. Hence we set out to determine whether C1q could bind to PS exposed on murine POS following NaIO_3_-induced outer BRB breakdown. Five hours after NaIO_3_ administration, C1q was present on a subset of PS positive POS (Fig. 3a,b, PS is detected using annexin V). C3 and C4 immunoreactive POS identified as rhodopsin-positive, were detected within 5 hours of NaIO_3_ treatment and diminished by 72 hours (Fig. 3c–f). Sub-optimal fixation conditions required to retain specificity of the detecting antibodies for the complement C3 and C4 antigens does not allow great detail of the POS. While low immunoreactivity for C1q, C3 and C4 was occasionally detected in the ONL and outer plexiform layer (OPL) of the retina and in the lumen of the retinal blood vessels, complement immunoreactivity was predominantly found in the outer layers of the retina, overlapping with rhodopsin immunostaining of the POS layer. Based on these results we propose that loss of the outer BRB secondary to NaIO_3_ administration is followed by transient exposure of POS to serum proteins that initiate CP and AP complement activation.

### Complement activation is required for the recruitment of mononuclear phagocytes to the outer retina

**Figure 4:**
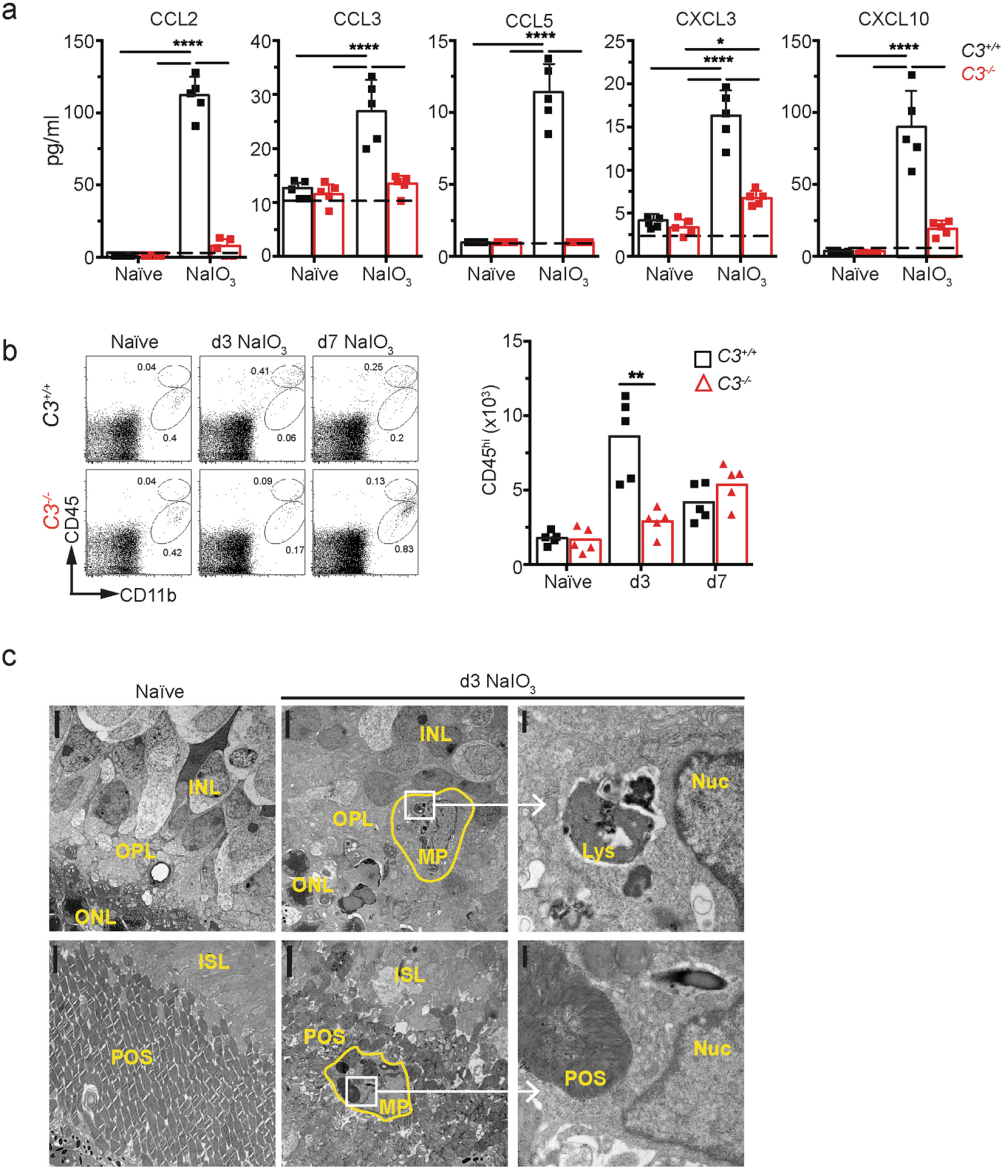
Complement C3 activation on photoreceptor outer segments results in recruitment of pathogenic mononuclear phagocytes. (**a**) Chemokine levels measured in retina lysates of *C3*^+/+^ and *C3*^−/−^ mice 24 hours after NaIO_3_, n = 5. Dashed line = limit of detection. Naïve = not NaIO_3_ treated *p < 0.05 and ****p < 0.001 by 2-way ANOVA with Tukey’s comparisons test. (**b**) Flow cytometry of CD11b^+^CD45^hi^ (recruited MP) vs CD11b^+^CD45^lo^ (microglia) cells in naïve mice, and in mice 3 and 7 days after NaIO_3_ administration, n = 5. Naïve = not NaIO_3_ treated. **p < 0.01, Mann-Whitney 2-tailed test. (**c**) Electron microscopy of retinas from a naïve mouse (left panels) or 3 days post NaIO_3_ administration (center and right panels). No MPs were present in naïve retinas. After NaIO_3_, MPs are located in the OPL (outer plexiform layer, upper panels) and photoreceptor segment layer (PSL, lower panels). Right upper panel is a close-up of a MP in the OPL that lacks POS in phagosomes or late lysosomes (Lys) while the lower right panels show a MP in the PSL layer that contains intact POS with multiple membrane stacks within a phagosome. Nuc = nucleus. Scale bar = 5 μm (2 left panels) and 0.5 μm (right panel).

To determine if complement activation on POS alters the local inflammatory milieu in the retina, we harvested the neural retina of *C*3^+/+^ and *C3*^−/−^ mice 1 day following NaIO_3_ administration to analyze protein levels of inflammatory mediators. The leukocyte chemokines CCL2, CCL3, CCL5, CXCL3 and CXCL10 were 2- to 5-fold increased in *C3*^+/+^ compared with *C3*^−/−^ retina 1 day following NaIO_3_ treatment (Fig 4a). Since these chemokines are important leukocyte chemo-attractants^47^, we next asked whether complement was associated with the migration of leukocytes to the outer retina layers following NaIO_3_ injection. In naïve mice, flow cytometry of the neural retina identified a single population of CD11b^+^CD45^l^° MPs resembling resident microglia (Fig. 4b, left panel)^48,49^. In mice treated with NaIO_3_ for 3 and 7 days, a population of CD11b^+^CD45^hi^ MPs was identified (Fig. 4b, middle and right panels). The number of CD45^hi^ MPs was significantly decreased in *C3*^−/−^ compared with *C3*^+/+^ mice 3 days following NaIO_3_ treatment. To study the cellular characteristics of the MPs localized to the outer retina layers in more detail, we performed electron microscope (EM) analysis of retinas from naïve and NaIO_3_ treated mice. MPs were absent in the OPL and PSL (photoreceptor segment layer) of naïve mice (Fig. 4c, left panels, n = 2). In retinas of mice 3 days following NaIO_3_ treatment, MPs in the OPL did not contain POS in phagosomes or lysosomes (based on the analysis of 3 MPs per mouse, n = 2; Fig. 4c, upper panels) while all MPs recruited to the photoreceptor inner- and outer segment layer contained several phagocytosed POS in their cytoplasm (based on the analysis of 9–10 MPs per mouse, n = 2) (Fig. 4c, lower panels).

### C3 activation increases POS phagocytosis and results in loss of functional photoreceptors

**Figure 5:**
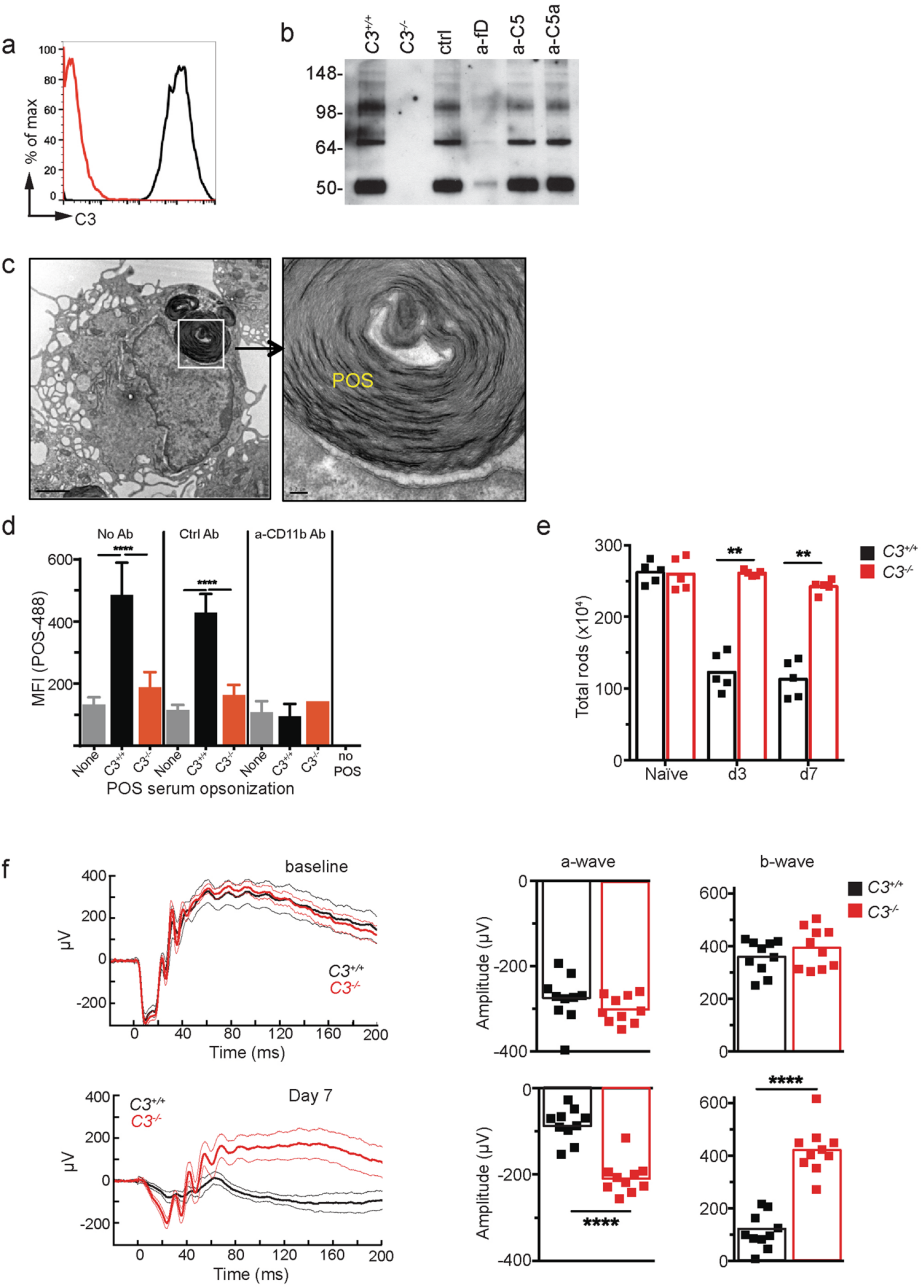
C3 activation is required for CR3-mediated phagocytosis of POS and photoreceptor loss. (**a**) iC3b opsonization on serum-exposed POS as shown by flow cytometry. (**b**) iC3b opsonization on murine C3 sufficient (*C3*^+/+^ and all Ab treatments) or deficient (*C3*^−/−^) serum-exposed POS as shown by Western blot. anti-CFD antibody (a-fD) partially blocks C3 opsonization on POS, with no effect by control, a-C5a or a-C5. (**c**) EM of a single thioglycolate-elicited peritoneal macrophage 30 minutes after incubation with bovine POS *in vitro*. Right panel shows a close-up of a POS with intact membranes inside a phagosome. Scale bar = 5 μm (left panel) and 0.5 μm (right panel). (**d**) Phagocytosis of fluorescently labeled POS by macrophages was increased by C3 opsonization and was dependent on CR3. No Ab, isotype control Ab (Ctrl) or anti-CD11b Ab (a-CD11b) was added to the macrophage culture prior to fluorescently labeled-POS. ***p < 0.001 and ****p < 0.0001 by 2-way ANOVA with Tukey’s multiple comparisons test. Error bars indicate SD of triplicate repeats. (**e**) Flow cytometry analysis of rods in *C3*^+/+^ and *C3*^−/−^ mice 3 and 7 days after NaIO_3_ administration, n = 5. Naive = not NaIO_3_ treated. (**f**) Dark-adapted ERG recordings of *C3*^+/+^ and *C3*^−/−^ mice 7 days after NaIO_3_ administration, n = 10. Thick lines are means and thin lines are 95% CI. Experiments were repeated at least twice with similar results. (E) and (F): **p < 0.01 or ****p < 0.0001, Mann-Whitney 2-tailed test. Experiments were repeated at least twice with similar results.

We next determined whether complement activation stimulated phagocytosis of POS by the MPs *in vitro*. Bovine POS incubated with *C3*^+/+^ mouse serum had covalently bound iC3b (Fig. 5a,b), a ligand for complement receptor 3 (CR3). iC3b was absent on POS incubated with serum from *C3*^−/−^ mice and was inhibited in the presence of anti-CFD antibody (a-fD). As expected, C3 opsonization of POS was unaffected by isotype control (ctrl), anti-C5 (a-C5) or anti-C5a (a-C5a) Ab treatment. Thioglycolate-elicited MP phagocytized intact POS *in vitro* (Fig. 5c,d). Efficient phagocytosis of POS was dependent on the presence of C3 in the opsonizing serum and CD11b, the alpha chain of the macrophage receptor for iC3b (CR3; Fig 5d). In summary, complement activation on POS is associated with an increased presence of MPs in the photoreceptor inner- and outer-segment layer and increased opsonization of POS with iC3b. Based on *in vitro* studies, phagocytosis of POS by MPs is dependent on iC3b opsonization and CR3 activation and inhibited by a-fD.

Consistent with these results, FACS analysis indicated significant sparing of rods on day 3 and day 7 following NaIO_3_ treatment in *C3*^−/−^ compared to *C3*^+/+^ mice (Fig. 5e). In order to assess photoreceptor function, we recorded ERGs in dark-adapted *C3*^+/+^ and *C3*^−/−^ mice at baseline and on day 7. While baseline a- and b-wave amplitudes were similar in *C3*^+/+^ and *C3*^−/−^ mice, *C3*^−/−^ mice showed significantly improved a- and b-wave amplitudes 7 days following NaIO_3_ administration (Fig. 5f), indicative of improved rod function^50^. In summary, blocking complement activation shows significant reduction in inflammatory monocyte recruitment along with physical and functional rescue of photoreceptors following NaIO_3_-induced RPE cell loss.

### Mononuclear phagocyte recruitment and photoreceptor degeneration require C5a

**Figure 6:**
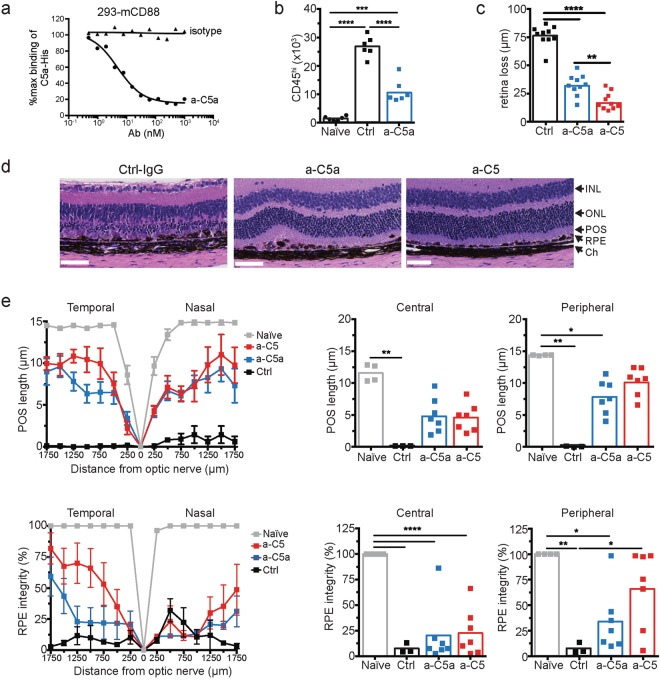
C5a is required for photoreceptor loss. (**a**) Anti-C5a (a-C5a) inhibits C5a binding to C5aR on the surface of 293 cells transfected with C5aR (CD88). (**b**) Flow cytometry analysis of CD11b^+^CD45^hi^ mononuclear phagocytes in the neural retina of mice treated with control or C5a neutralizing Abs 3 days following NaIO_3_ administration, n = 6. (**c**) Effect of C5a and C5 blocking Abs or isotype control Abs (Ctrl) on retina degeneration as measured by SD-OCT 7 days after NaIO_3_ administration. (**d**) Representative H&E stained sections of the central retina 7 days after NaIO_3_ administration. (**e**) POS length and RPE integrity measured in horizontal sections along the temporal-nasal axis of the mouse retina. Error bars indicate ± SEM, n = 3–4 naïve and Ctrl, n = 7 a-C5a and a-C5. Naïve = not NaIO_3_ treated. Scale bar = 10 μm. Central is 500 μm from optic nerve and periphery is 500–1750 μm. Experiments were repeated at least twice with similar results. *p < 0.05, **p < 0.01, ***p < 0.001, ****p < 0.0001 by 1-way ANOVA with Tukey’s multiple comparisons test. INL = inner nuclear layer; ONL = outer nuclear layer; POS = photoreceptor outer segments; RPE = retinal pigmented epithelia; Ch = choroid.

C3bC3b homodimers or C3bC4b heterodimers form the scaffold of the C5 convertases that process C5 into two effector molecules, the chemo-attractant C5a^51^ and the cytolytic MAC initiator C5b^52^. In the NaIO_3_ model, administration of an antibody (a-C5a) that specifically blocks binding of mouse C5a to its receptor C5aR/CD88 (Fig. 6a) reduced the number of CD11b^+^CD45^hi^ MP cells in the retina by ~60% of control antibody-treated mice at 3 days following NaIO_3_ administration (Fig. 6b). While inhibiting C5a activity significantly protected retina and reduced MP recruitment, we asked whether inhibiting generation of both C5a and C5b, the latter required for MAC formation, could provide retinal protection superior to C5a blockade alone. As demonstrated by SD-OCT imaging, retina loss was reduced by ~60% in mice treated with the C5a blocking Ab (Fig. 6c). An antibody that blocks mouse C5 activation (a-C5) and therefore prevents the formation of both C5a and C5b (clone BB5.1^53^) did provide significantly improved protection of the retina over blocking C5a alone. Morphometric analysis demonstrated that a-C5a protected POS in the central and peripheral retina, but a-C5 treatment did not provide improved protection compared to a-C5a antibody treatment alone (Fig. 6d,e). Hence, C5a is critical in inducing POS loss following C5 activation in the retina. Central RPE integrity was not attenuated by C5 blockade, whereas a-C5 protected peripheral RPE. While complement C5 activation may not play a role in the initial NaIO_3_ toxicity in RPE, it may affect subsequent complement C5-dependent spreading of RPE cell death into the periphery^54^.

### Peripheral blood monocytes, but not resident microglia, are required for C5a-dependent photoreceptor loss

**Figure 7:**
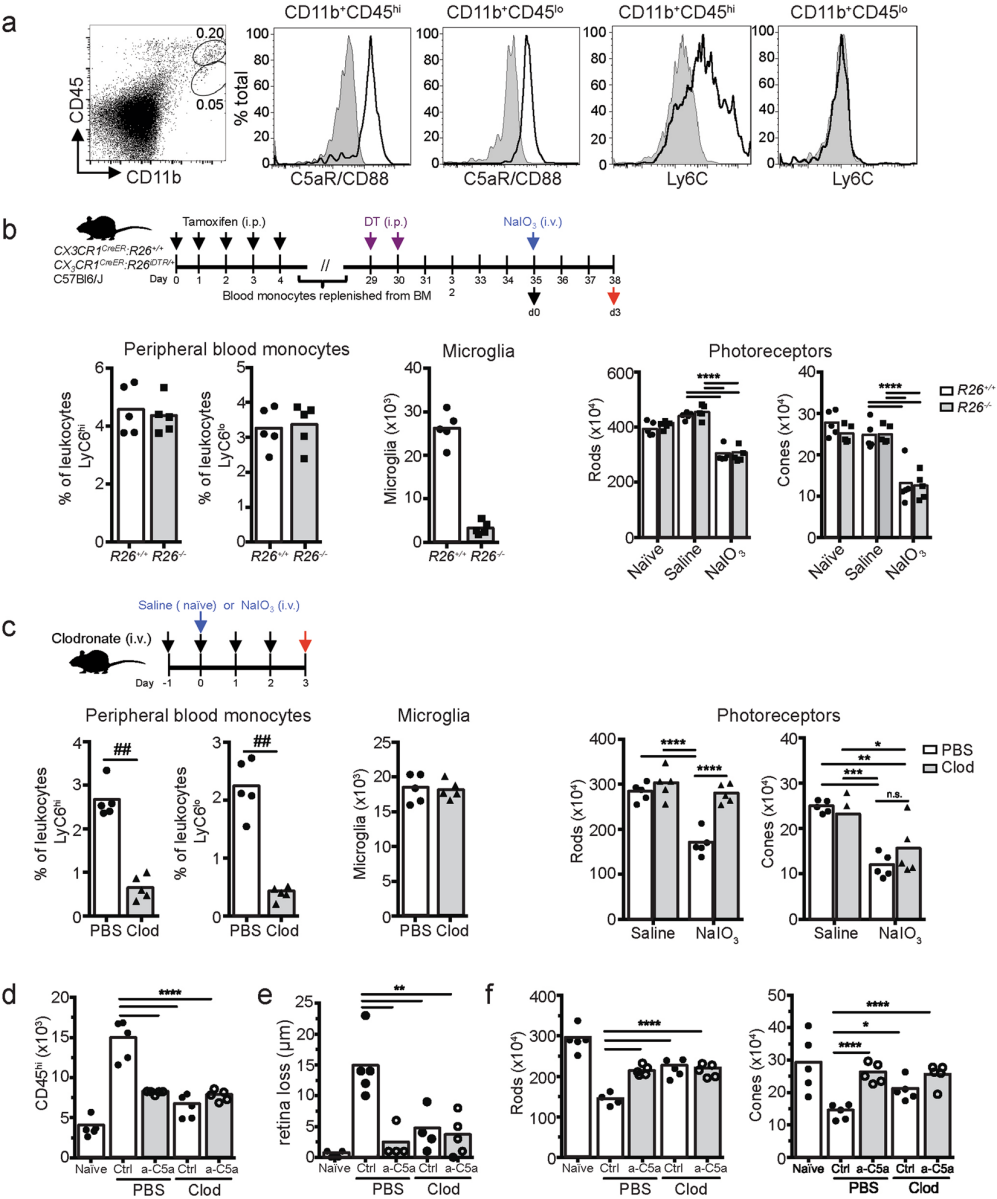
Peripheral blood monocytes, but not resident microglia cells, are required for C5a mediated photoreceptor degeneration. (**a**) C5aR (CD88) and Ly6C expression on CD11b^+^CD45^lo^ and CD11b^+^CD45^hi^ cells in the retina following NaIO_3_ administration. Shaded area indicates isotype control. (**b**) Flow cytometry following diphtheria toxin (DT) treatment showed no change of circulating Ly6C^hi^ and Ly6C^lo^ monocytes but DT depleted resident retina microglia (left 3 graphs n = 5). Right two graphs show no change in rod or cone photoreceptor loss due to NaIO_3_ after DT treatment. Naïve = no DT or NaIO_3_ treatment. Saline = DT treatment with saline control injection, NaIO_3_ = DT treatment with NaIO_3_ injection. ^##^p < 0.01 by Mann-Whitney 2-tailed test and ****p < 0.0001 by 2-way ANOVA with Tukey’s multiple comparisons test. (**c**) Flow cytometry of circulating Ly6C^hi^ and Ly6C^lo^ monocytes, resident microglia and photoreceptor rods and cones following treatment with PBS- or clodronate-containing liposomes (n = 5). ^##^p < 0.01 by Mann-Whitney 2-tailed test and *p < 0.05, **p < 0.01, ***p < 0.001, ****p < 0.0001 by 2-way ANOVA with Tukey’s multiple comparisons test. (**d**) Flow cytometry of retinal CD11b^+^CD45^hi^ cells of C57BL/6 J mice treated with PBS or clodronate liposomes, and isotype (Ctrl) or neutralizing a-C5a Abs. (n = 5). (**e**) SD-OCT analysis of retina loss after NaIO_3_ administration (n = 5). (**f**) Flow cytometry of photoreceptor rods and cones (n = 5). Naïve = no NaIO_3_. *p < 0.05, **p < 0.01, ****p < 0.0001, analyzed by 2-way ANOVA, not including naïve condition. All data were obtained at 3 days after NaIO_3_ administration. Experiments were repeated at least twice with similar results.

Given that the C5a receptor CD88 is expressed on both CD11b^+^ CD45^l^° Ly6C^-^ retinal microglia cells and CD11b^+^ CD45^hi^ Ly6C^+^ retinal MPs following RPE cell injury (Fig. 7a), we next determined the contribution of microglia versus MPs to photoreceptor cell loss. Microglia were depleted using diphtheria toxin treatment of mice expressing the diphtheria toxin receptor (DTR) under control of the *CX3CR1* promoter^55^(*CX*_*3*_*CR1*^*CreER*^*:R26*^*iDTR/*+^mice). By pulsing with tamoxifen and treating with diphtheria toxin (DT) 30 days later, 91% of the resident microglia cells were depleted, while numbers of circulating CD115^+^Ly6C^hi^ and CD115^+^Ly6C^lo^ peripheral blood monocytes were unaffected (Fig. 7b). Depletion of microglia prior to NaIO_3_ treatment did not protect photoreceptor rods or cones. In a second approach, we selectively depleted circulating CD115^+^Ly6C^hi^ and CD115^+^Ly6C^lo^ peripheral blood monocytes using clodronate-encapsulated liposomes while not affecting microglia numbers (Fig. 7c). This resulted in significant sparing of rod photoreceptors, but not cones, 3 days after NaIO_3_ treatment. Clodronate depletion of peripheral blood monocytes caused a significant reduction of retinal CD11b^+^CD45^hi^ MP, indicating that peripheral blood monocytes are a major source of the retinal CD11b^+^CD45^hi^ MPs (Fig. 7d). Inhibiting C5a did not further reduce MP numbers in clodronate-treated mice. Finally, both clodronate depletion and C5a blockade reduced retina loss (Fig. 7e) and spared rods and cones, with no additive effect of C5a blockade and clodronate treatment (Fig. 7f). Hence, in this preclinical model of retinal degeneration, C5 activation critically contributes to photoreceptor and RPE cell loss through C5a-mediated recruitment of pathogenic peripheral blood Ly6C^hi^CD88^+^CCR2^+^ monocytes to the outer layers of the retina.

### Complement activation through both classical and alternative complement pathway contribute to inflammation and retinal degeneration

**Figure 8:**
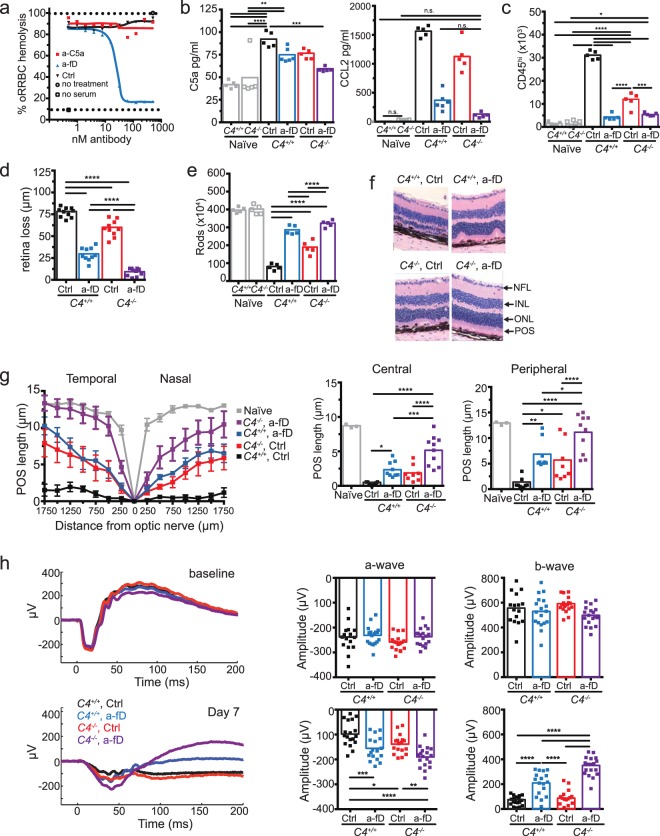
Both classical/lectin and alternative complement pathways contribute to retinal degeneration. (**a**) Inhibition of complement-mediated rabbit red blood cell hemolysis by mouse serum using anti-mouse CFD blocking antibody (a-fD). (**b**) *C4*^+/+^ and *C4*^−/−^ mice were treated with anti-CFD (a-fD) or isotype control (Ctrl) Ab to address the contribution of CP/MBL and AP to inflammation and retinal degeneration. C5a and CCL2 concentrations were measured in retinal homogenates 24 hours after NaIO_3_ administration, n = 5–6, Naïve = no NaIO_3_. For CCL2 graph not significant (n.s.) comparisons are indicated, otherwise comparisons are significant. (**c**) Flow cytometry analysis of CD11b^+^CD45^hi^ cells in the neural retina 3 days after NaIO_3_ treatment in *C4*^+/+^ or *C4*^−/−^ treated with Ctrl or a-fD (n = 5). Naïve = no NaIO_3_ (**d**) Effect of complement inhibition on retinal loss measured by SD-OCT 7 days after NaIO_3_ administration, n = 10. (**e**) Quantification of rod photoreceptors 7 days after NaIO_3_ administration in *C4*^+/+^ or *C4*^−/−^ treated with Ctrl or a-fD (n = 5). Naïve = no NaIO_3_ (**f**) Effect of complement inhibition on retinal morphology in H&E stained horizontal sections through the retina, 7 days after NaIO_3_ administration. (**g**) Complement inhibition and POS length measured in horizontal sections along the temporal-nasal axis of the mouse retina 7 days after NaIO_3_ administration in *C4*^+/+^ or *C4*^−/−^ treated with Ctrl or a-fD. Naïve = no NaIO_3_. Error bars indicate ± SEM, n = 8–10. (**h**) Dark adapted ERG a-wave and b-wave amplitudes 7 days following NaIO_3_ treatment in *C4*^+/+^ or *C4*^−/−^ treated with Ctrl or a-fD (n = 5). Naïve = no NaIO_3_, n = 17–18. Data from two experiments were pooled *p < 0.05, **p < 0.01, ***p < 0.001, or ****p < 0.0001, 2-way ANOVA with Tukey’s multiple comparisons test. All experiments were repeated at least twice.

C5 is a common downstream target of complement activation initiated through the CP, MBL, and AP (Fig. 2b). To determine the contribution of these three complement pathways to C5 activation and retina degeneration, we used a mouse deficient in C4 to eliminate CP and MBL pathway activation^56^. Systemic pharmacological blockade of the AP was achieved using an antibody that neutralizes CFD (a-fD, Fig. 8a), an enzyme critical for AP activation and currently a therapeutic target in GA^57^. While both CP/MBL and AP activation contributed to C5a production, AP activation was the main driver for CCL2 production and CD11b^+^CD45^hi^ MP recruitment to the retina (Fig. 8b,c). Combined loss of C4 and pharmacological blockade of CFD was required for optimal rescue of photoreceptor rods as well as POS length (Fig. 8d–g). Consistent with sparing of photoreceptors, combined blockade of CP/MBL and AP was required to maximally improve a- and b-wave amplitudes (Fig. 8h). Combined blockade of CP/MBL and AP was superior to inhibition of C5 activation alone (Compare Fig. 8g with 7e), consistent with a role for C3 opsonization, independent of the C5 convertase, in promoting POS phagocytosis. Hence, these results demonstrate a critical role for complement C3 convertases of the CP/MBL and AP in the induction of photoreceptor and RPE cell loss secondary to NaIO_3_ -induced BRB disruption.

### Complement activation following laser-induced blood-retina barrier disruption causes POS loss

**Figure 9:**
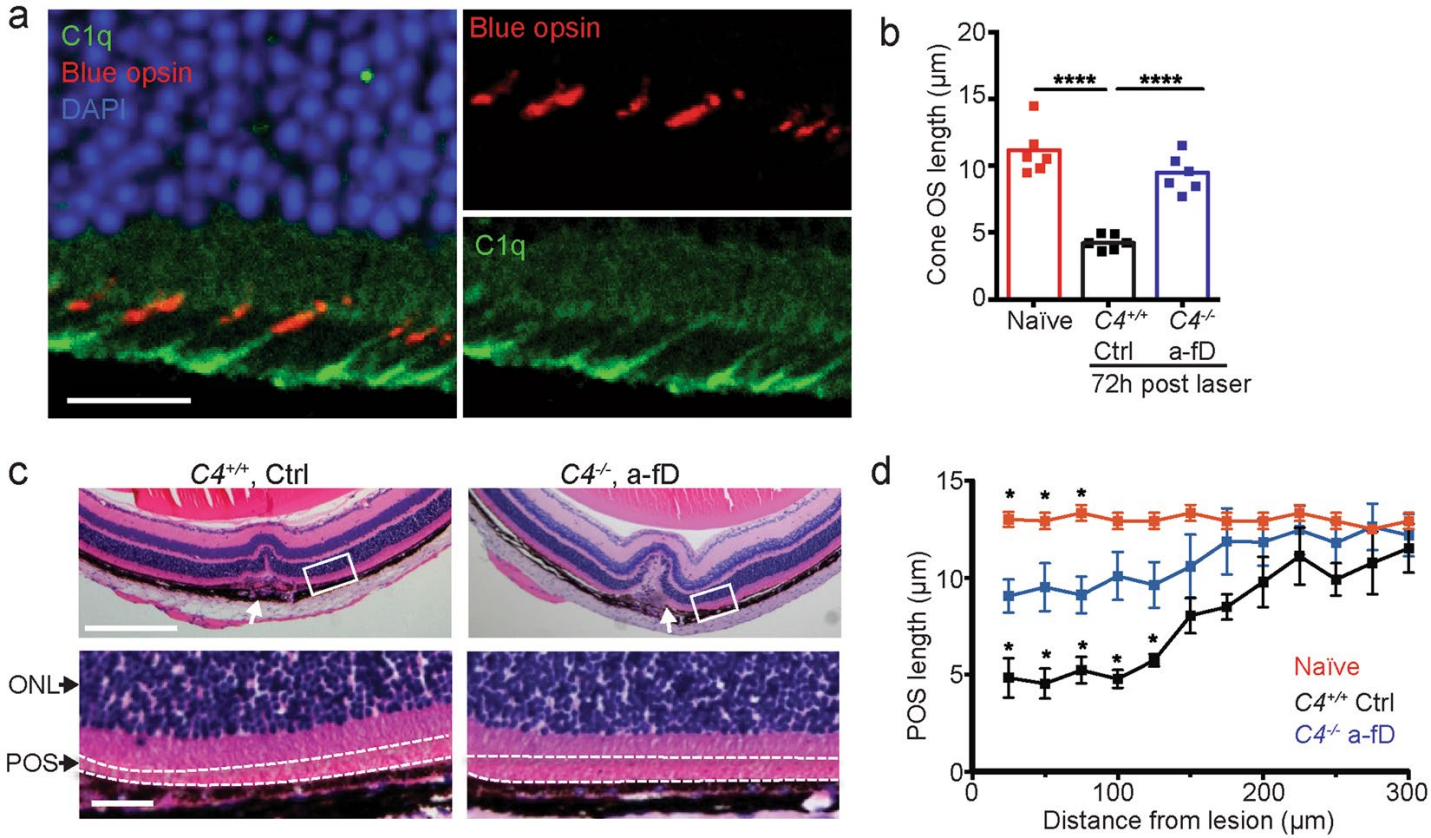
Systemic inhibition of CFD and deletion of C4 protects POS from laser-induced BRB disruption. (**a**) C1q immunoreactivity at the interface of blue opsin-positive cone outer segments and the apical border of the RPE, 24 hours after laser injury. Images are taken 300 μm nasally of the laser injury. (**b**) Cone outer segment length in naïve (*C4*^+/+^ no laser), *C4*^+/+^ mice treated with isotype Ab (Ctrl) and *C4*^−/−^ mice treated with a-fD, 72 hours after BRB disruption with a laser. ****p < 0.0001, 1-way ANOVA with Tukey’s multiple comparisons test. (**c**) H&E stained section of a lesion site 24 hours after laser injury (white arrows) indicating POS length (close-up of boxed inset, lower panels) in *C4*^−/−^ mice treated with a-fD compared to *C4*^+/+^ mice treated with control Ab. The POS layer is located between the dashed lines. (**d**) POS length measured as a function of distance from the lesion site or, in the naïve group, the distance from a site that corresponds anatomically to the site of the lesion in the treated group. Scale bar = 25 μm. *p < 0.05 Mann-Whitney test vs *C4*^−/−^ a-fD treatment group, n = 5. ONL = outer nuclear layer; POS = photoreceptor outer segment.

Bruch’s membrane damage and RPE cell loss are frequently associated with advanced AMD^34^. In order to determine if complement activation is implicated in loss of POS independent of NaIO_3_, we induced BRB disruption by laser injury. Complement immunoreactivity on POS and RPE was observed within a 200 μm radius from the lesion site, 24 hrs after laser-induced injury (Fig. 9a). Both blue opsin-positive (cones) and –negative (rods) photoreceptors showed immunoreactivity for C1q at the POS-RPE interface. Following laser injury, cone outer segment length was reduced by ~50% 3 days following laser injury, but almost completely recovered in C4^−/−^ mice treated with a-fD (Fig. 9b). POS length was reduced by 8 μm, but restored by >50% in C4^−/−^ mice treated with a-fD blocking Ab (Fig. 9c,d). These results demonstrate that laser-induced BRB disruption, similar to NaIO_3_-induced damage, results in complement activation on POS and complement-mediated loss of POS in the retina adjacent to the laser injury.

## Discussion

In this study, we demonstrate that complement C3 and C4 proteins are absent in the lesion area of GA postmortem eyes except for occasional immunoreactivity in retinal and choroidal vasculature. In a subset of donor eyes with GA, complement C3 and C4 staining was localized to POS in the transition zone were pathological changes in choriocapillaris, RPE and photoreceptors have been described^28^ as well as in an area ~500 µm peripheral of the transition zone. Given that the transition zone temporally precedes the expansion of the GA lesion area, it is conceivable that complement activation on POS contributes to GA lesion area growth. Complement activation on otherwise intact POS may lead to recruitment of inflammatory monocytes that in turn affect the RPE and neural retina survival. The association of complement C3 and C4 activation with POS in a subset of human donor eyes was consistent with the increased sensitivity of POS to complement activation *in vitro* and *in vivo*. Hence, complement activation may contribute to cellular changes at earlier stages of the disease that precede more prominent histopathological features characteristic of the GA lesion and transition zone.

Mouse models of NaIO_3_-induced RPE cell loss and laser-induced RPE injury were used as “pathway models” to define the molecular and cellular basis of complement-mediated retinal injury *in vivo*. Maximal retinal protection in these preclinical models was achieved by blocking both the CP/MBL and AP pathways, upstream of iC3b-mediated phagocytosis of POS and C5a-dependent monocyte recruitment to the outer retina. Hence, targeting the AP pathway alone may not be sufficient to protect the retina from complement attack. Using approaches to selectively deplete either circulating monocytes or resident microglia, we determined that inflammatory monocytes are required, but microglia are dispensable for complement-dependent retinal cell loss in the NaIO_3_ model of retina degeneration. These results are compatible with the observation that systemic inhibition of CFD or C5 with neutralizing antibodies is sufficient to protect the retina in models of RPE disruption. An increased number of CD163^+^ myeloid cells have been found in the outer retinal layers of eyes diagnosed with intermediate and advanced AMD compared to non-AMD cases^44^. Further studies are required to determine if these myeloid cells are pathogenic and if they originate from resident microglia migrating towards the outer retinal layers or from circulating monocytes that infiltrate the retina from peripheral blood.

Studies in rodent models of dry AMD point to a critical role of complement regulation through soluble (CFH) or cell surface-expressed (CD46) inhibitors in preserving retinal homeostasis^17,20,21^. This is consistent with the low threshold for complement activation on POS that lack prominent negative regulators of complement activation (CD46, CD55) but expose PS capable of initiating classical pathway complement activation^38,58^. Studies that have investigated a role of complement in neovascular AMD implicated the anaphylatoxins C3a and C5a in angiogenesis either directly through C3aR and C5aR activation on endothelial cells^59,60^, or indirectly through recruitment of VEGF-producing MP to the retina^18^. In our study, we demonstrate that complement activation, secondary to outer BRB disruption with a laser, results in an early loss of POS prior to choroidal neovascularization. Complement risk alleles predispose to both the neovascular and dry forms of AMD^9,61^, consistent with complement being implicated in early pathophysiological processes, including the formation of drusen and subretinal drusenoid deposits, that have been reported to precede both neovascular AMD and GA^5,24^. The source of complement observed in the retina of a subset of GA cases needs further investigation but may be systemic in origin and the result of transient disruption of the blood-retinal barrier as evidenced by the presence of sub-retinal choroidal neo-vessels observed in a sub-population of patients diagnosed with GA^6,7^.

Based on human genetics and preclinical studies a critical role for complement alternative pathway activation in retinal degeneration has been proposed^11,12,16,17^. We now identified a potentially important contribution of CP activation in photoreceptor cell loss. In addition, we demonstrate an important role for C3 convertases in addition to C5 convertases in retinal degeneration. Our observation of classical pathway contribution to retinal degeneration is consistent with recent findings in neurodegenerative diseases where complement activation initiated through the classical pathway has been implicated in schizophrenia^62^. In studies on Alzheimer’s disease, C1q binding and C3-opsonization of presynaptic terminals led to pruning of synapses and was closely associated with human disease manifestation^63,64^. Hence, synaptic pruning in neurodegenerative disease and photoreceptor loss associated with macular degeneration may share a common molecular and cellular basis.

As the preclinical models described in this study do not recapitulate important pathophysiological features associated with GA, including the formation of sub-RPE deposits and drusenoid deposits^5,28^, caution should be observed when extrapolating the efficacy results obtained in the described “pathway” models to therapeutic intervention in human disease. In this context, it is important to note that two identically designed phase 3 clinical studies (NCT02247531 and NCT02247479, ClinicalTrials.gov) demonstrated that lampalizumab, a factor D blocking antibody, failed to inhibit GA lesion growth, despite positive results in a phase 2 clinical trial^65^. Similarly, clinical trials inhibiting C5 systemically^66^ or locally (NCT02534909) failed to meet their pre-specified endpoint. A phase 2 clinical study with APL-2, an inhibitor of the central complement protein C3 (NCT02503332), met its primary endpoint by showing a significant reduction in GA lesion growth, suggesting that targeting all complement pathways at the C3 convertase level may be required for maximal efficacy. The lack of effect of AP complement inhibition on GA lesion area growth despite the high disease risk carried by genetic variants in complement genes, may indicate that complement contributes to pathological processes that precede the advanced stage of the disease. Therapeutic intervention at an earlier stage of the disease will likely be required for complement modulators to be successful in the clinic.

## Methods

### Human samples

Postmortem human donor eyes were obtained from the Lions Eye Institute for Transplant and Research in Tampa, Florida. Clinical records and a family questionnaire were obtained for all donors. The stage and type of AMD disease (neovascular or GA) was determined based on donor ophthalmic history (see Supplemental Table 1 for donor information) combined with histologic evaluation using the Alabama age-related macular degeneration grading system for donor eyes^67^. Out of a total of 42 eyes of donors previously diagnosed with GA and/or neovascular AMD in one or both eyes, 19 donor eyes were selected for further study based on the presence of histopathological features reminiscent of GA^28,29^ and the absence of retina detachments or retina tears due to processing of the tissue. Human eyes were fixed in Davidson’s fixative (3:3:2:1 volumes ethanol, distilled water, neutral buffered formalin and glacial acetic acid, respectively) for 24 hours then transferred to 70% ethanol. Average death-to-preservation time for eyes from AMD donors was 5 hours and 40 minutes and that for control donors was 5 hours and 20 minutes.

### Immunohistochemistry on human tissue

Various antibodies reactive with C3 were tested for antigen specificity in cell pellets. CHO cells were dissociated with trypsin, coated with rabbit-anti-hamster lymphocyte (Accurate Chemical), opsonized in NHS or C3 depleted human serum (Cell Signaling Technology) and processed similar to the human donor eyes. Rabbit anti-C3, clone EPR19394 (Abcam, Cambridge, MA) was identified as specific based on results from cell pellet tests.

Immunofluorescence of complement proteins was performed on transverse sections through the macular area. Eyes were paraffin embedded and 4 μm sections covering the retina and optic nerve were cut along the vertical meridian of the globe as described previously^68^. Sections were prepared with DAKO Target Retrieval Solution (Agilent, Santa Clara, CA) then blocked for endogenous peroxidase and biotin activity. Dual immunofluorescence staining for C3 and Rhodopsin, clone RET-p1 (Santa Cruz Biotechnologies, Dallas, TX) was performed sequentially with PowerVision- Poly HRP anti-mouse and PowerVision Poly-HRP anti-rabbit (Leica Biosystems, Buffalo Grove, IL), respectively, for detection. C3 was visualized by TSA- Alexa Fluor 555 and Rhodopsin by TSA -Alexa Fluor 647 (ThermoFisher Scientific, Carlsbad, CA). An elution step in Target Retrieval was performed between sequences to eliminate cross detection. For dual staining, anti-C4d clone C4D204 (Novus Biologicals, Littleton, CO) and anti-rhodopsin, clone EPR7996 (Abcam, Cambridge, MA) were used. Anti-rhodopsin was detected with biotinylated donkey anti-rabbit IgG (Jackson Immunoresearch, West Grove, PA), followed by Streptavidin- HRP and visualized with TSA-Alexa Fluor 555. After elution in Target Retrieval, mouse anti-C4d was detected with PowerVision- Poly HRP anti-mouse, followed by TSA Alexa Fluor 647. For CFB immunostaining, sections where stained with monoclonal Ab clone MCA2647 (Serotec) as described previously^35^. PLVAP immunohistochemistry was performed as described previously^68^. For C3 co-staining with red/green opsin (Millipore Sigma, Danvers, MA), rabbit anti- red/green opsin was detected with PowerVision Poly-HRP anti-rabbit with TSA-Alexa Fluor 488. Following elution, C3 was detected with biotinylated donkey anti-rabbit IgG followed by Vectastain Elite ABC- HRP (Vector Laboratories, Burlingame, CA) and visualized with TSA-Alexa Fluor 555. All samples were DAPI stained before adding Prolong Gold (ThermoFisher Scientific, Carlsbad, CA) and cover-slipped. Naïve mouse IgG1 (Pharmingen), naïve rabbit IgG (Cell Signaling Technologies) and naïve mouse IgG2a (Pharmingen) were used as isotype controls for C4, C3 and CFB immunohistochemistry, respectively. Fluorescence images were obtained using a Hamamatsu nanozoomer XR system for fluorescent images, and a HT system for brightfield images. The systems are equipped with a time delay integration scan camera. Peak excitation and peak emission wavelengths (nm) of the fluorophores are 359/461 for DAPI, 433/475 for CFP (purple), 499/520 for Alexa Fluor 488 (green), 553/565 for Alexa Fluor 555 (red) and 653/668 for Alexa Fluor 647 (yellow).

### Mice

All animals were held under sterile pathogen-free conditions. *C3*^−/−^ mice were generated as described^69^ and backcrossed for at least 10 generations to C57BL/6 J mice. *C4*^−/−^ mice were generated as described^56^. Mice were re-derived and crossed for 6 generations to C57BL/6 J mice. CX3CR1^CreER^ mice were obtained from Dr. Dan Littman and Dr. Wen-Biao Gan^55^ and crossed to Rosa26^iDTR^ (C57Bl6/J) mice obtained from the Jackson Laboratory to generate *CX3CR1*^*CreER/*+^:*Rosa26*^*iDTR/*+^ *and CX3CR1*^*CreER/*+^:*Rosa26*^+/+^ mice (controls)^55^. Littermate wild-type controls were used for all experiments. C57BL/6 J from Jackson Laboratory were used in experiments that did not use genetically deficient mouse strains.

### POS opsonization and *in vitro* analysis

Bovine photoreceptor outer segments (InVision BioResources) isolated as described^70^ were washed 2 times in DMEM/2.5% sucrose, aliquoted and frozen at −80 °C until needed. POS were washed 2x in gelatin veronal buffer (GVB) with Ca^2+^/Mg^2+^ (GVB++, Complement Technology). 5 × 10^6^ POS were incubated in 20% normal (NHS) or complement deficient human serum (Complement Technology) in 100 μl for 12 minutes at 37 °C. The reaction was stopped with 100 μl GVB/EDTA (Complement Technology) +1x FUT-175 (BD). POS were washed 2x in FACS buffer (PBS/0.5% BSA/2 mM EDTA) and stained with either 1 μg/ml mouse-anti-C9 neo-epitope (Hycult clone aE11), mouse-anti-C4d (Quidel), or 1:400 FITC-conjugated goat-anti-C3 (MP Biomedicals) followed by anti-mouse-A647 (Jackson Immunoresearch). Flow cytometry was performed on a FACS Calibur (BD) and analyzed in FlowJo. Complement regulatory proteins and annexin V were assessed by flow cytometry with rat-anti-CD46 (Serotec), rat-anti-CD55 (Hycult), rat-anti-CD59 (Serotec) or annexin V (Life Technologies), data collected on FACS Calibur (BD) and analyzed with FlowJo software (FlowJo).

Bovine POS were incubated with human serum depleted of various complement proteins. Serum from *C3*^−/−^, *C4*^−/−^, or *Rag2*^−/−^ mice was collected in serum separator tubes (BD), flash frozen and stored at −80 °C until needed. 5 × 10^6^ POS with 20% serum in GBV++ were incubated for 45 min at 37 °C and reaction stopped with GVB/EDTA/Fut-175. C3 and C4 were detected on POS by flow cytometry using goat-anti-mouse-C3 -FITC (1:400, MP Biomedical) and rat-anti-mouse C4 (0.75 ug/ml, Hycult clone 16D2) followed by anti-rat −647 (Jackson ImmunoResearch). C3 fragment opsonization on POS was visualized by Western blot. 5 × 10^6^ POS were lysed in Laemmli’s sample buffer with 2% β2-mecraptoethanol, boiled, and separated on 4–20% Tris-glycine gel (Invitrogen) and C3 fragments detected with HRP-conjugated goat-anti-mouse-C3 (1:5,000, MP Biomedicals). Opsonization of C57BL/6 J mouse retina cells were performed on digested retina as previously described^68^ and lymphocytes from whole blood. Briefly, retina from a single eye was isolated and digested with papain (Worthington Biomedical) and washed in PBS/2 mM CaCl_2_/0.5 mM MgCl_2_. 2 × 10^6^ retina cells in 100 μl were opsonized with 20 ul mouse serum for 30 minutes at 37 °C. 100 ul ice cold PBS/10 mM EDTA was added and cells washed 2x with PBS/0.5% BSA/2 mM EDTA. Whole blood from C57BL/6 J mice was collected in heparin, 100 μl aliquoted in round bottom FACS tubes, and RBCs were lysed with 2 ml ACK lysis buffer. Cells were washed and serum opsonized as retina cells. Retina and blood cells were stained for flow cytometry analysis as POS, with retina cells stained with violet Live/Dead cell stain (ThermoFisher) and lymphocytes were stained with anti-CD3 PE-Cy7 (clone 145-2C1, eBioScience) and propidium iodide to gate out dead cells.

### NaIO_3_ induced retinal degeneration

Male C57BL/6 J mice aged 8–10 weeks were intravenously injected with 20 mg/kg body weight of sodium iodate (NaIO_3_) (Sigma) or saline control on day 0. The dosage of NaIO_3_ was chosen based on previous dose titration experiments assessing RPE damage by fundus imaging and retina thickness change by SD-OCT (data not shown). Antibody treatment (a-C5, a-C5a, a-CFD) was given daily i.p. at 1 mg/mouse starting 2 days before NaIO_3_ injection. This dose maintained trough Ab concentrations in the serum which were at least 10 fold-higher than the IC90 of a-CFD in a hemolytic assay using undiluted serum (Fig. 8a). Tissue was collected for analysis at 5 hours, 1, 3 or 7 days after NaIO_3_ administration.

### Staining of live murine photoreceptor outer segments

After CO_2_ asphyxiation, eyes were enucleated and placed in HBSS with Ca^2+^ and Mg^2+^ for dissection of the anterior chamber and removal of the RPE/sclera eyecup. After isolating the retina, four radial cuts were made in order for the retina to lie flat and the retina was placed outer segment side facing up in a 48-well plate containing FluoroBrite DMEM with anti-C1q Ab (1:100, ThermoFisher) labeled with quantum dots (SiteClick Molecular Probes) and incubated at 37 °C for 30 minutes. Ab solution was gently removed and retina was washed for 1 minute each in FluoroBrite DMEM. Finally, the well was filled with HBSS plus Mg^2+^ and Ca^2+^ with annexin V conjugated with Alexa Fluor 594 (1:1000, ThermoFisher) for 20 minutes before mounting the retina outer segment side up in FluoroBrite DMEM on a microscope slide with a glass coverslip. Slides were imaged with a Leica SPE upright confocal microscope.

### Immunohistochemistry on mouse tissue

For albumin, C1q, and blue opsin staining, mouse eyes were fixed in Davidson’s fixative for 24 hours, transferred to 70% ethanol, then processed in an automated paraffin tissue processor. Paraffin embedded samples were sectioned axially at 5 μm and cut through the midline of the eye for NaIO_3_ or through the laser-induced lesions.

For C3, C4, and rhodopsin staining of mouse retina sections, eyes were enucleated and fixed in methacarn for 30 minutes at room temperature then cryoprotected in a 10%, 20% 30% sucrose gradient, 12 hours each step. Eyes were briefly washed in PBS before embedding in tissue freezing medium (General Data Healthcare) with 70% ethanol cooled over dry ice. Before cryosectioning, samples were trimmed until the lens was visible and able to be removed with fine tipped forceps and OCT medium was added to fill in the lens space. Samples were then cryosectioned at 12 μm and dried at room temperature before storing at −20 °C.

Paraffin sections were rehydrated with 5 minute washes in 100% xylene (2x) followed by 100% ethanol (2x), 90% ethanol (2x), 70% ethanol (2x) before finally washing in PBS for 15 minutes. Frozen sections were thawed at room temperature before staining. Both types of samples were stained in the same manner with the following protocol: samples were blocked for 1 hour in 10% donkey serum/0.3% triton X-100 in PBS, stained for 2 hours at room temperature with rabbit-anti-C3 (1:100, Dako), rat-anti-C4 (1:100, clone 16D2, ThermoFisher), mouse-anti-rhodopsin (1:100 clone 1D4, Santa Cruz), rabbit-anti-blue opsin (clone 5407, Millipore) or goat-anti-mouse albumin (1:200, clone sc-46293, Santa Cruz) in 0.1% Triton X-100/PBS, washed 15 minutes in PBS for two rounds, then incubated in secondary antibody for 1 hour at room temperature in anti-rabbit-488, anti-rat-488, anti-mouse-555 or anti-goat-A488 (1:1000, Jackson ImmunoResearch) in 0.1% triton X-100 in PBS for 1 hour. After secondary antibody incubation, the slides were washed for two rounds of 15 minutes in PBS before being mounted in Mowiol 4–88 (Sigma) and stored at 4 °C until imaged with a Nikon A1R microscope. Fluorescence intensity (FI) of complement staining was obtained using FIJI software measuring integrated density. Background FI was subtracted by the average of three readings in areas of the slide that had no tissue.

### ELISA and Luminex

Retina protein was collected in 300 μl cell lysis buffer (Cell Signaling) with protease inhibitors (Roche Complete, FUT-175, phosphatase inhibitor cocktail II and PMSF [Cell Signaling]) in 500 μl screw cap tube (Sarstedt). Tissue was homogenized using two 3 mm tungsten beads with a Qiagen Tissuelyser II, 3 minutes at 30 Hz and centrifuged for 15 minutes at 4 °C at 18,000 × g. Lysate was aliquoted and stored at −80 °C. ELISAs for mouse C5a (RayBiotech) and CCL2 (DuoSet, R&D Systems) were performed according to manufacturers instructions and read on Spectramax Plus (Molecular Devices). Luminex analysis on 5 retina lysates was performed using Milliplex 32-plex panel (EMD Millipore). We only reported chemokines that were detected in retina samples.

### Flow cytometry of retinal cells

Flow cytometry was performed as previously described^68^. Briefly, retina from a single eye was isolated and digested with papain (Worthington Biomedical). Total retinal cells were quantified with a standard concentration of 6 μm Fluoresbrite® YG microspheres (Polysciences). Surface antigens CD11b-PE-Cy7 (clone M1/70, BD), CD90.2-APC (clone 53-2.1, BD), and CD45-A700 (clone 30-F11, Biolegend) were stained and live cells gated by propidium iodide exclusion. Intracellular antigens rhodopsin (rods, clone 1D4, EMD Millipore) and cone arrestin (cones, EMD Millipore) were directly conjugated with PE-Cy7 or PE, respectively, with Ab conjugation kits (Abcam). Dead cells were detected by violet fixable dye (ThermoFisher) prior to fixation (IntraPrep Permeabilization kit, Beckman Coulter). Flow cytometry was done on an LSRFortessa using FACSDiva software (BD) and data analyzed with FLowJo software. Total numbers of cells were calculated by multiplying percentage of cell type to total live cells.

### Electron Microscopy

Tissues and cultured cells were first fixed in modified Karnovsky’s fixative (2% paraformaldehyde and 2.5% glutaraldehyde in 0.1 M sodium cacodylate buffer, pH 7.2) and then post-fixed in 2% aqueous osmium tetroxide (EM Sciences, Hatfield, PA) for 2 h followed by incubation in 0.5% uranyl acetate for 1 h. The samples were then washed and dehydrated through a series of ethanol (50%, 70%, 90%, 100%) followed by a final propylene oxide step and embedded in Eponate 12 (Ted Pella, Redding, CA). Ultrathin sections (80 nm) were cut with an Ultracut microtome (Leica), stained with 0.2% led citrate and examined in a JEOL JEM-1400 transmission electron microscope (TEM) at 80 kV. Digital images were captured with a GATAN Ultrascan 1000 CCD camera.

### Phagocytosis

Peritoneal MPs were elicited from C57BL/6 J (Jackson) by i.p. injection of 1 ml 4% thioglycolate (Sigma) for 96 hours and harvested with a 10 ml lavage of ice-cold DMEM/5 mM EDTA. After 2 washes, 1.5 × 10^6^ cells/well in DMEM/10% FBS/1x penicillin/streptomycin were seeded in a 12-well dish overnight. Bovine POS were labeled with AlexaFluor-488 by washing 9 × 10^8^ POS in 0.1 M sodium bicarbonate buffer, adding 1 vial protein labeling kit AlexaFluor-488 (Molecular Probes), and rotating in the dark for 1 hour in 2 ml volume. POS were washed 2 times in DMEM/2.5% sucrose. Labeled POS were opsonized with serum obtained from *C3*^+/+^ or *C3*^−/−^ mice. Adherent macrophages were washed 2x with PBS and replaced with DMEM/2.5% sucrose media. 50 μg/ml anti-CD11b (M1/70, BD) or rat IgG2b isotype (ctrl, BD) was added 10 minutes before adding 15 × 10^6^ POS for 60 minutes at 37 ^o^C. Cells were washed 2x with PBS, trypsinized for 10 minutes then scrapped off for flow cytometry. Cells were washed in FACS buffer, flow cytometry performed on FACS Calibur and analyzed on FlowJo software.

### Spectral Domain Optical Coherence Tomography (SD-OCT)

Retinal thickness was measured by SD-OCT using the Spectralis HRA + OCT system (Heidelberg Engineering). Mice were anesthetized by intraperitoneal injection of ketamine (70–80 mg/kg body weight) and xylazine (15 mg/kg body weight). Pupils were dilated with drops of Tropicamide Ophthalmic Solution USP 1% (Akorn). Genteal moderate eye drops (Alcon) were applied bilaterally to prevent corneal dehydration during procedure. Horizontal volume scans through the region dorsal-temporal from the optic nerve (superior quadrant) were used to evaluate the retina thickness. Total retina thickness was defined as the width from the nerve fiber layer to the RPE/choroid layer on cross-sectional images with custom written image segmentation routines in Matlab (MathWorks).

### Electroretinography (ERG)

ERG recordings were performed with the Espion2 electrophysiology system (Diagnosys LLC). Mice were dark-adapted overnight before ERG recording, and all procedures were performed under dim red light. Mice were anesthetized and their pupils dilated as described above. Body temperature was maintained using a homeothermic plate and held at 37 °C. A reference electrode was inserted subcutaneously through the forehead and a ground electrode was inserted subcutaneously in the lumbar region. A gold-ring electrode (animal electrode 0.5 mm ø 3 mm) (LKC) was placed on the corneal surface of each eye. A drop of Goniovisc Hypermellose Ophthalmic Demulcent Solution 2.5% (HUB Pharmaceuticals) was applied on the cornea to establish an electrical contact between the cornea and the electrode, and to maintain corneal moisture during procedure. The mice were placed on a platform covered with a ColorDome light stimulator. Eyes were stimulated with 25 cd.s/m^2^ white light and results are the average of five flashes with 1 minute recovery time between flashes. Signals were bandpass-filtered at 0.15–1000 Hz and sampled at 2 kHz. Between animals, electrodes were cleaned using ethanol wipes then rinsed with sterile PBS. After ERG, ophthalmic ointment was topically applied on the cornea to prevent desiccation. All of the recorded data points were analyzed using custom Matlab code with a-wave amplitude measured from the baseline to the trough of the a-wave while b-wave amplitude from the trough of the a-wave to the peak of the b-wave. Responses to 3–5 flashes of light stimulation were averaged.

### Antibodies

Anti-mouse factor D clone 3D6 was generated in a hamster by immunization with mouse factor D protein. The antibody CDR domains were subsequently grafted on a mouse IgG1 backbone. The binding affinity (K_*D*_) of the anti factor D antibody is 3.8 nM. Anti-C5a clone 46 was generated by immunizing C5-deficient A/J mice with recombinant mouse C5a. Antibodies were screened for binding to the C5a portion of C5, for their ability to inhibit C5a binding to its receptor and for their ability to inhibit C5a-induced Akt phosphorylation in the J774A.1 mouse macrophage cell line. Mouse IgG1-anti-C5 clone BB5.1F8 has been characterized elsewhere^53^.

### Morphometry

Each retina section was scored in 250 μm increments from the optic nerve. Pixel length of the POS was measured in four 50 μm segments within each 250 μm region before averaging those measurements and converting the length to microns. The POS was identified in H&E sections by its lighter hue than the photoreceptor inner segments. Retina was analyzed for RPE integrity in H&E sections that included the optic nerve. RPE was scored 0–100 in increments of 5, 0 = complete destruction and 100 = complete protection. Naïve RPE was characterized by even distribution of pigment and thickness of the RPE layer. Scoring was devised as follows: 80–100, RPE was still a continuous layer but had thinned and condensed pigment; 60–80, partial breaks in RPE continuity and some pigmented cells in the outer segment (OS) layer was observed; 40–60, RPE layer was discontinuous in several areas of the retina, with several pigmented cells in the OS; 20–40, significant disruption of the RPE layer throughout the retina; 0–20, complete disruption of the RPE layer and several pigmented cells observed between the choroid and the outer nuclear layer. Each retina was scored by analyzing four 50 μm segments and averaging them within a 250 μm section.

### Depletion of peripheral blood monocytes and microglia

Control or clodronate encapsulated liposomes (Encapsula Nanosciences) were injected intravenously daily starting 1 day before NaIO_3_ administration. Clodronate depletion of peripheral monocytes/macrophages was confirmed by flow cytometry. To deplete microglia, *CX3CR1*^*CreER/*+^:*Rosa26*^*iDTR/*+^ *and CX3CR1*^*CreER/*+^:*Rosa26*^+/+^ mice (controls) were given five doses of 80 mg/kg of Tamoxifen (Sigma) in sesame oil by intraperitoneal injection (i.p.) for five consecutive days. Thirty days after the first Tamoxifen dosing, mice were given i.p. two doses of 2 ug diphtheria toxin (DT) (Sigma) for two consecutive days. Blood monocytes and retinal microglia were analyzed by FACS 6 days after the first DT dosing. Mice were injected with NaIO_3_ on day 6 after the first DT dosing and photoreceptors were analyzed 3 days after NaIO_3_ injection by FACS as described^68^. Blood was collected by cardiac puncture and erythrocytes were removed by ACK lysis buffer (Life Technologies). Cells were resuspended in PBS/2% FBS/2 mM EDTA and Fc blocked (BD) and stained for CD115-APC (clone AFS98, eBiosciences) or Ly6C-FITC (clone AL-21, BD).

### Rabbit red blood cell hemolysis

Rabbit red blood cells (RRBCs, Complement Technology) were washed 2x in GVB and resuspended in GVB/0.1 M EGTA/0.1 M MgCl_2_. Antibodies were diluted in GVB, mixed with C57BL/6 J serum (20%, 100 μl final volume), then 40 × 10^6^ RRBCs were added for 45 minutes at 37 °C. 220 μl GVB/40 mM EDTA was added to stop the reaction, then centrifuged at 1600 rpm for 8 min and 200 μl supernatant was removed and OD read at 412 nm. Percent hemolysis was calculated from RRBCs lysed in untreated serum (=100%).

### Laser-induced retinal blood barrier disruption

C57BL/6 J mice were anesthetized with a cocktail of ketamine (75 mk/kg) and Xylazine (7.5 mg/kg) through an intraperitoneal injection. Pupils were dilated with 1% tropicamide ophthalmic solution USP (Akorn, Lake Forest, IL). Choroidal neovascularization was induced with a 532 nm laser system (OcuLight GL Photocoagulator, Iridex, Mountain View, CA). A coverslip was placed on the surface of the mouse cornea as a contact lens to view and focus on the retina. Laser spots were created in each eye using a Zeiss SL30 slit lamp system with a laser spot size of 100 μm, 120 mW power and 100 ms duration. Three burns in eye at the 3, 6, and 12 o’clock positions around the optic nerve were made, each burn 2–3 optic disk diameters (about 200–300 μm) from the optic nerve in both the left and right eye. The presence of a bubble at the time of laser application confirmed the sufficient rupture of Bruch’s membrane. Cases of subretinal hemorrhage induced by the laser were excluded from the analysis. Animals received analgesic twice daily (buprenorphine, 0.05 mg/kg) IP starting the day of the procedure. The first dose was administered at the time of anesthesia, prior to the procedure.

### Statistics

All data, unless otherwise indicated, were graphed and statistical analysis was performed in Prism 6 software (GraphPad). Normal distribution was determined using the D’Agostino-Pearson normality test. Normally distributed data was analyzed using parametric analysis of variance (ANOVA) with either Tukey’s multiple comparison test to compare multiple groups or Dunnet’s multiple comparison test when comparing one group against several others. Non-parametric data was analyzed by the Mann-Whitney test to compare two unpaired groups or the Kruskal-Wallis test with Dunn’s multiple comparisons test for multiple unmatched groups. P < 0.05 was considered significant.

### Study approval

The studies using human tissues were performed in accordance with FDA regulations and in accordance with the Eye Bank Association of America (EBAA) medical standards regarding utilization of human tissue. Written, informed consent was obtained from all donors who provided human samples. The protocols for these studies were approved by the Pharma Repository Governance Committee at Genentech that serves as the Genentech/Roche Institutional Ethical Committee to ensure that research on human samples stored in Genentech bio-repositories is performed in accordance with the subject’s informed consent and with global ethical guidelines. All animal experiments were approved by Genentech’s Institutional Animal Care and Use Committee and were performed according to the ARVO Statement for the Use of Animals in Ophthalmic and Vision Research.

### Data availability

The datasets generated during and/or analyzed during the current study are available from the corresponding author on reasonable request.

## Electronic supplementary material

Supplement
