# Publisher Correction: Classical and alternative complement activation on photoreceptor outer segments drives monocyte-dependent retinal atrophy

**DOI:** 10.1038/s41598-018-30162-w

**Published:** 2018-08-24

**Authors:** Kenneth J. Katschke, Hongkang Xi, Christian Cox, Tom Truong, Yann Malato, Wyne P. Lee, Brent McKenzie, Rommel Arceo, Jianhua Tao, Linda Rangell, Mike Reichelt, Lauri Diehl, Justin Elstrott, Robby M. Weimer, Menno van Lookeren Campagne

**Affiliations:** 1 Department of Immunology, Genentech, Inc, South San Francisco, CA 94080 USA; 2 Department of Translational Immunology, Genentech, Inc, South San Francisco, CA 94080 USA; 3 Department of Pathology, Genentech, Inc, South San Francisco, CA 94080 USA; 4 Department of Biomedical Imaging, Genentech, Inc, South San Francisco, CA 94080 USA

Correction to: *Scientific Reports* 10.1038/s41598-018-25557-8, published online 09 May 2018

In the original version of this Article, the author Menno van Lookeren Campagne was incorrectly indexed.

Additionally, in the original version of this Article, Figure 8g contains an incorrect key.

These errors have now been corrected in the HTML and PDF versions of this Article.

