# Supplementary material for: Classical and alternative complement activation on photoreceptor outer segments drives monocyte-dependent retinal atrophy
Source: Sci Rep. 2018 May 9;8:7348. doi: 10.1038/s41598-018-25557-8 (PMC5943270; doi:10.1038/s41598-018-25557-8)
Supplement: Supplementary file 1 — Supplement [file 41598_2018_25557_MOESM1_ESM.pdf]

**Supplementary dataset**

# Classical and alternative complement activation on photoreceptor outer segments drives monocyte-dependent retinal atrophy

Abbreviated title: A complement-monocyte axis drives progressive retinal atrophy


Kenneth J. Katschke Jr.[1], Hongkang Xi[1], Christian Cox[1], Tom Truong[2], Yann Malato[2], Wyne P. Lee[2], Brent McKenzie[2], Rommel Arceo[3], Jianhua Tao[3], Linda Rangell[3], Mike Reichelt[3], Lauri Diehl[3], Justin Elstrott[4], Robby Weimer[4] and Menno van Lookeren Campagne[1]

[1]Department of Immunology, [2]Translational Immunology, [3]Pathology and [4]Biomedical Imaging, Genentech, Inc., South San Francisco, CA 94080, USA

Corresponding author: Menno van Lookeren Campagne, menno@gene.com

## Supplemental Figure Legends

### Supplemental Figure 1: Visualization of photoreceptor atrophy in human donor eyes diagnosed with GA

(a) From left to right, top to bottom: Cross-section through a 70 year old female control donor eye (left panel) and an 82 year old female donor diagnosed with GA 2 years prior to death, an 88 year old female donor left eye and right eye diagnosed with GA 14 years prior to death and a 92 year old male donor diagnosed with GA 10 years prior to death. Rhodopsin-positive POS are shown in yellow and are absent in the atrophic zone. White arrowheads indicate the location of C3 (upper right image) and C4 (all other GA images) positive POS. Scale bar = 2.5 mm.

(b) Localization of C4 immunoreactivity (red) in the rhodopsin-positive POS layer (yellow) of an 89 year old male control donor eye (left panel) and an 82 year old female donor diagnosed with GA 2 years prior to death (right panel). White arrowheads indicate C4 positive POS, green arrowheads indicate C4 staining in the choriocapillaris. Scale bars = 50 μm.

### Supplemental Figure 2: Presence of CFB immunoreactivity in the photoreceptor outer segment layer of donor eyes diagnosed with GA

(a) Immunohistochemical detection CFB (red) in a 67 year old female control donor eye (left panel) and 82 (middle panel) and 88 (right panel) year old female donor eyes diagnosed with GA. CFB is detected in the choroid of all donor eyes, and in the photoreceptor outer segment layer of donor eyes diagnosed with GA.

(b) Immunohistochemistry results on two serial sections obtained from the GA donor eye shown in the middle panel of (a). In the left panel, the primary antibody has been replaced with a mouse

IgG2a isotype control antibody (isotype). The right panels show sections through human liver stained with a-CFB antibody or with isotype control antibody. GCL = granule cell layer, INL = inner nuclear layer, BM = Bruch's membrane. Scale bars = 50 µm (a, left panels in b) or 10 µm (right panel in b).

**Supplemental Figure 3: Specificity of C3 and C4 immunohistochemistry**

(a) Sections through a human 86 year old female donor diagnosed with GA. Upper panels: Immunohistochemical detection of C4 on rhodopsin$^{+}$ POS. Lower panels: Serial sections in which the primary antibody was replaced with a mouse IgG1 isotype control antibody (Isotype). Arrows point to POS which show C4 immunoreactivity in a section incubated with a-C4 mAb but not in a section incubated with isotype control Ab.

(b) Cross-sections through the same donor eye stained with an anti-C3 polyclonal Ab (right panel) or a rabbit isotype control antibody (Isotype, left panel). Thick arrows point to C3 immunoreactivity on POS in a section stained with anti-C3 pAb and the absence of immunoreactivity in a section from the same donor stained with an isotype control Ab.

(c) C3 immunostaining on sections cut through a pellet of CHO cells incubated with normal human serum (NHS, right panel) or human C3 depleted serum (left panel). Scale bar (all panels) = 100 µm

**Supplemental Figure 4: Complement opsonization of photoreceptors and CD3$^{+}$ lymphocytes.**

**(**a) Dissociated live retinal cells (dot plots on the left) were stained for cell-surface complement inhibitors CD46 and CD55 or stained for opsonized C3 and C4 following incubation with mouse

sera (histograms on the right). Upper histograms: Solid line is isotype control, shaded area is CD46 or CD55 staining. Lower histograms: Solid line represents cells incubated with $C3^{-/-}$ or $C4^{-/-}$ serum and shaded area represents cells incubated with wildtype serum.

(b) Mouse peripheral blood cells (dot plots on the left) were stained for cell-surface complement inhibitors CD46 and CD55 or stained for opsonized C3 and C4 following incubation with mouse sera (histograms on the right). Dot plots of live $CD3^{+}$ cells. Upper histograms: Solid line is isotype control, shaded area is CD46 or CD55 staining. Lower histograms: Solid line represents cells incubated with $C3^{-/-}$ or $C4^{-/-}$ serum and shaded area represents cells incubated with wildtype serum.

**Supplemental Figure 5. Choroidal neovascularization in a donor eye diagnosed with GA**

Cross-section through the right (OD) and left (OS) eye of an 82 year old female donor diagnosed with GA 2 years prior to death (same case as shown in Fig 1). PLVAP (green), a marker for fenestrated choroid vessels. Boxed sections in the left panels are enlarged in the right panels. Arrows in the right upper panel indicate $PLVAP^{+}$, sub-retinal neo-vessels. OD=oculus dexter (right eye), OS=oculus sinister (left eye), BM = Bruch’s membrane, Ch = choroid. Scale bar = 50 µm.

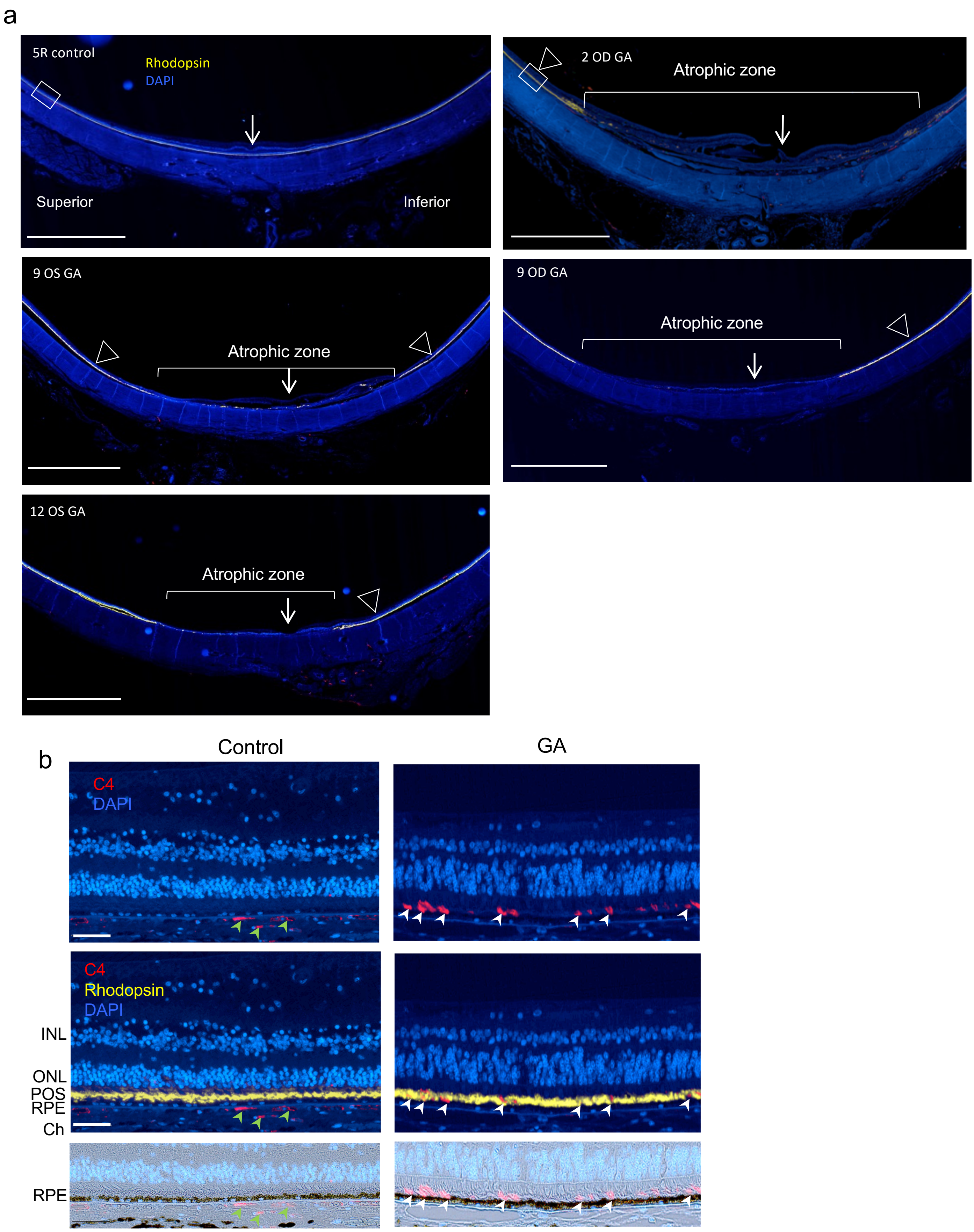


Supplemental Fig 1

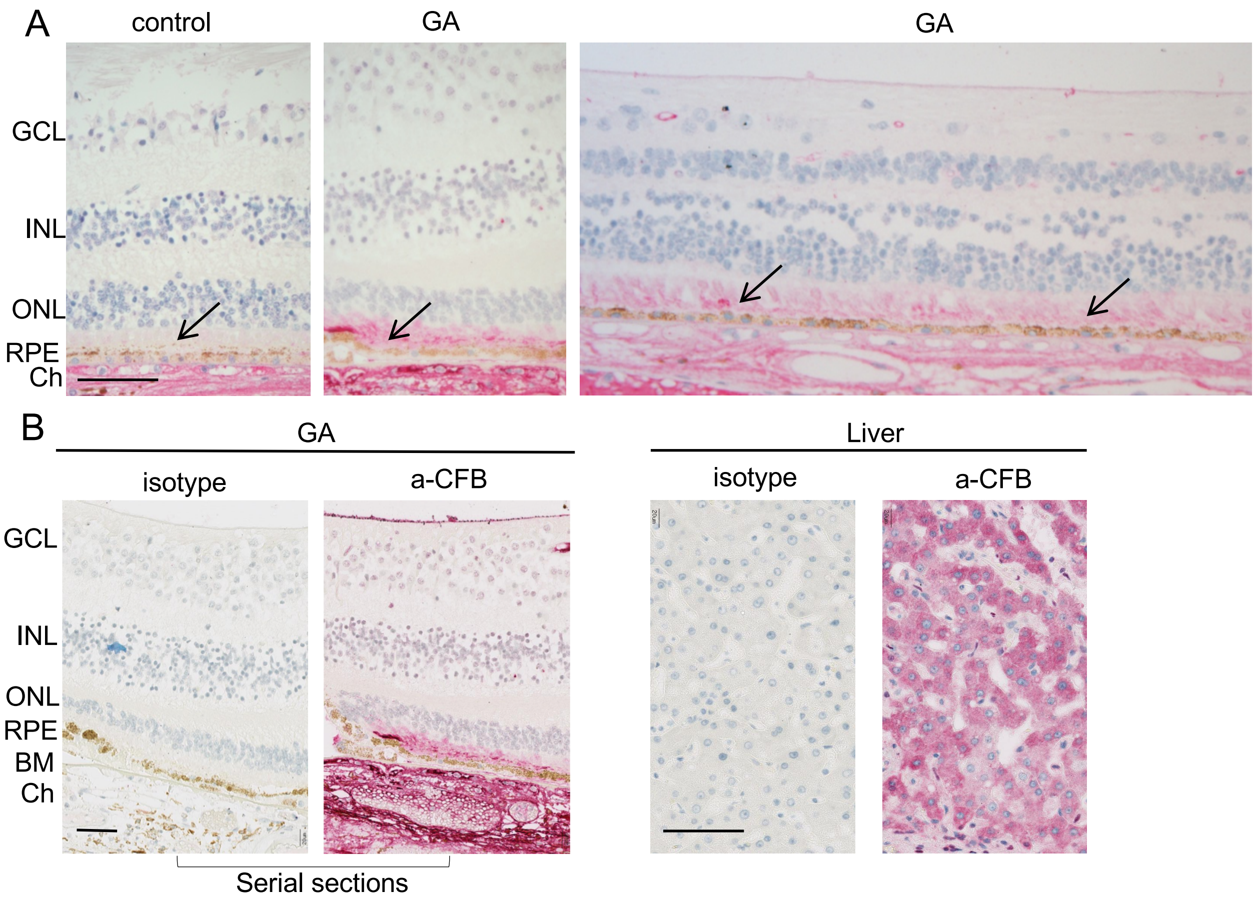


Supplemental Fig 2

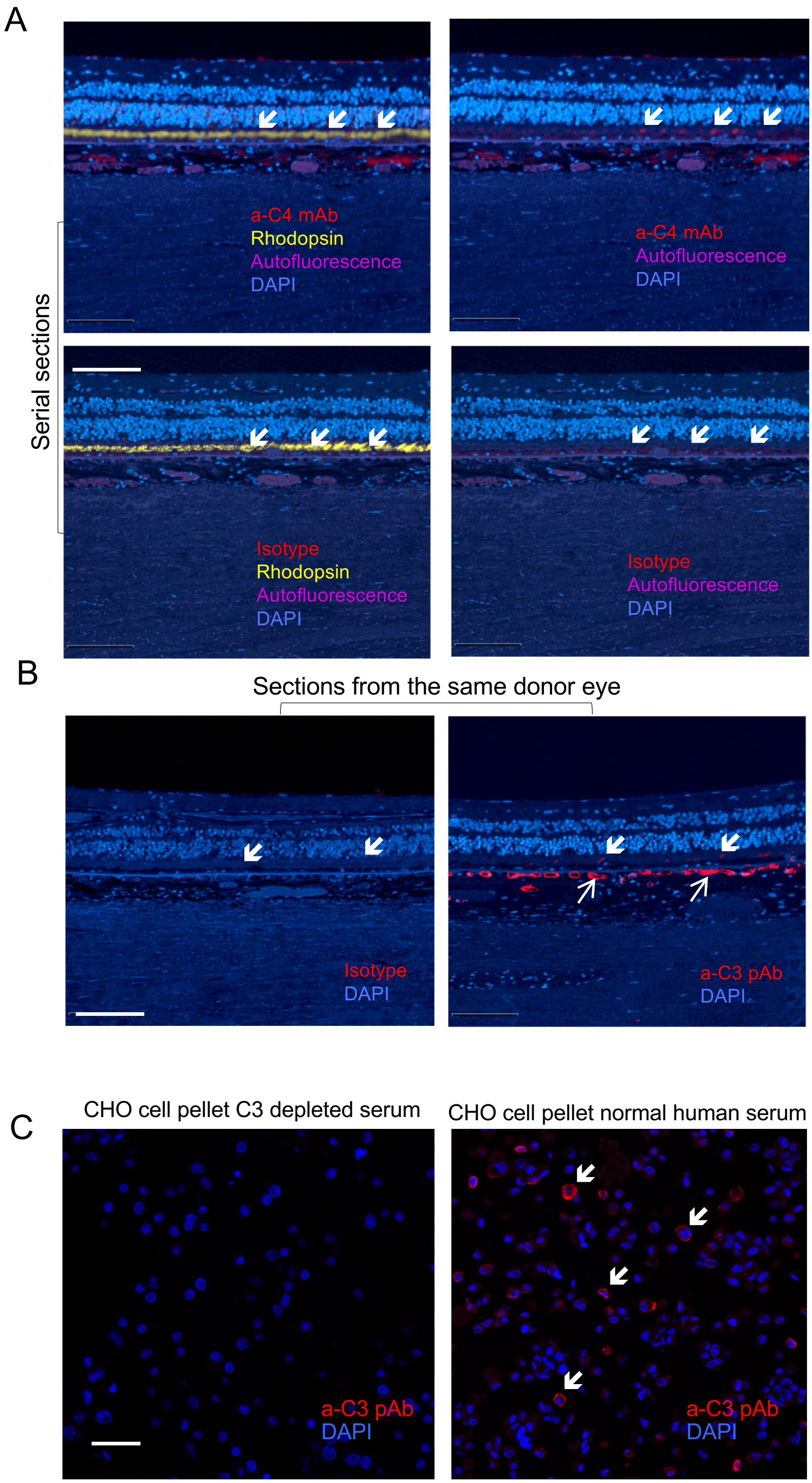


Supplemental Fig 3

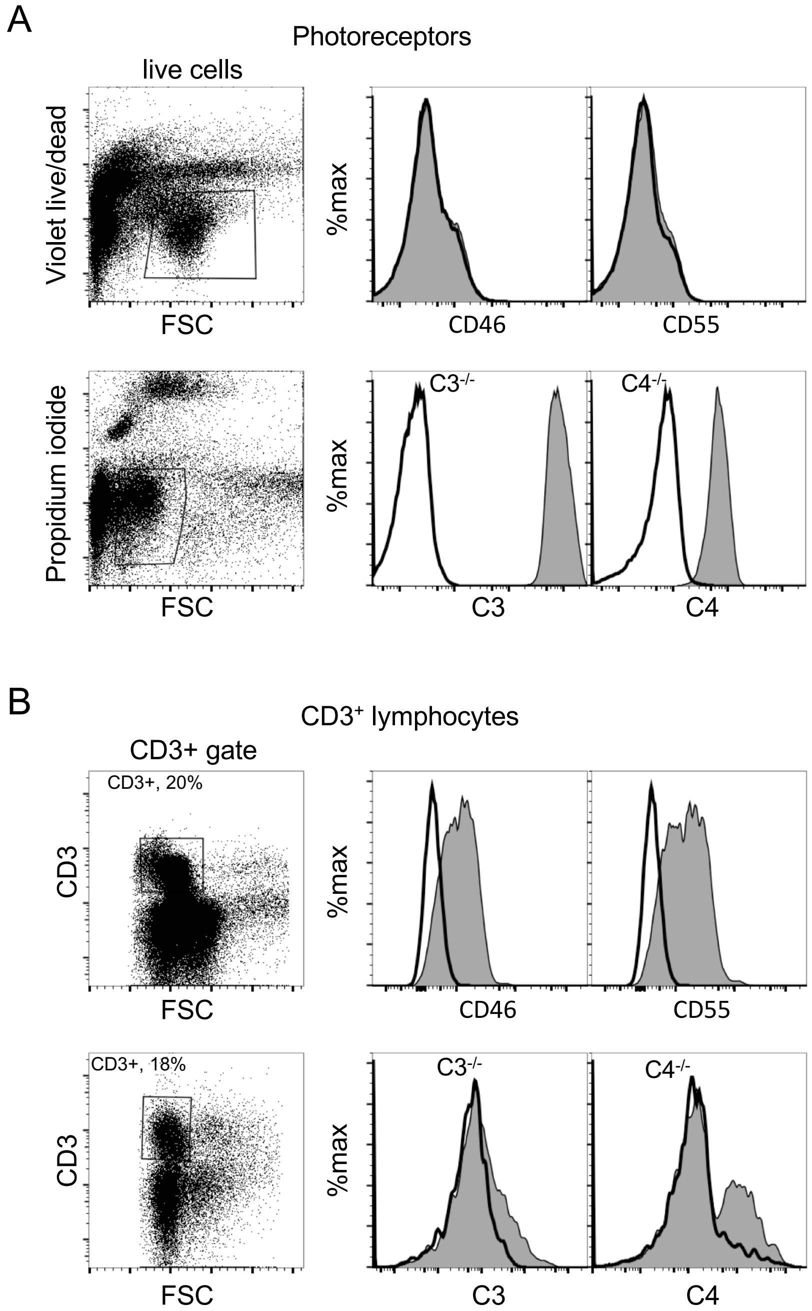


Supplemental Fig 4

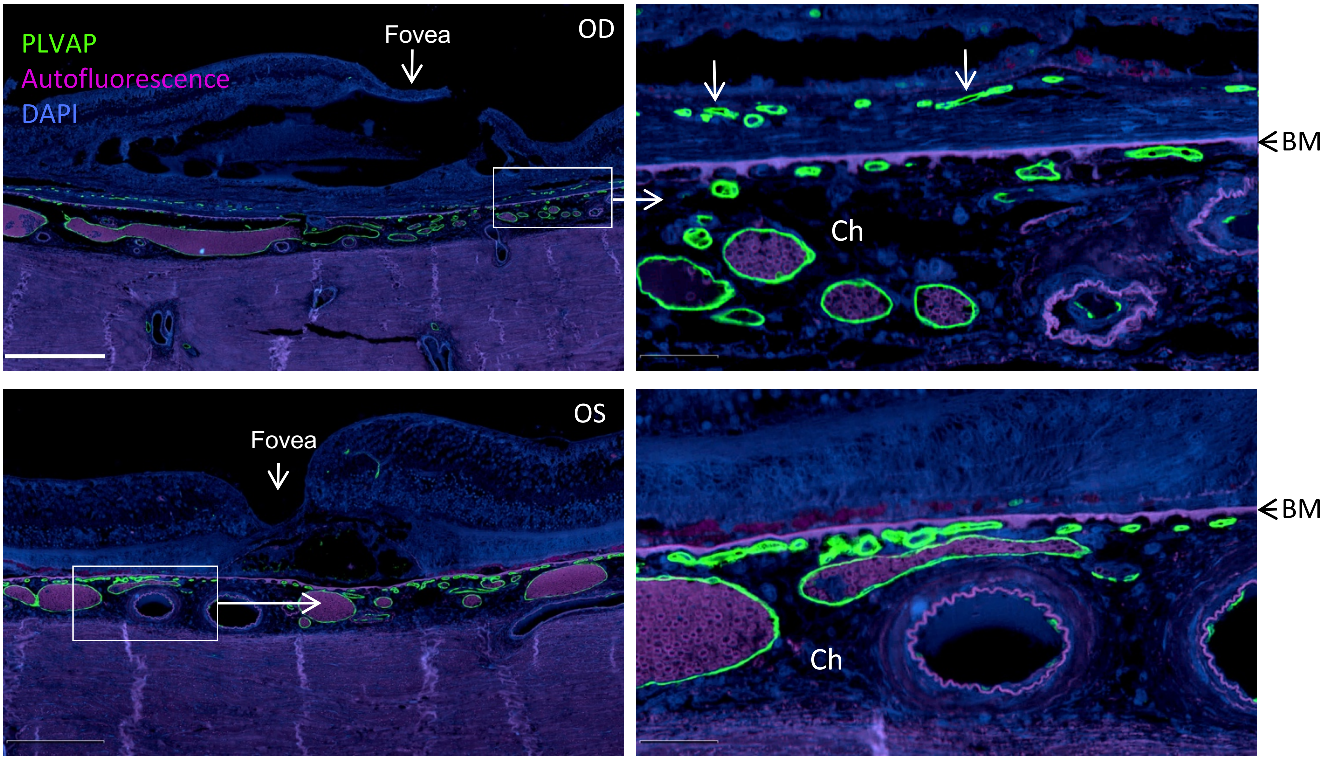


Supplemental Fig 5

Supplemental Table 1: Donor characteristics for CFB staining

| | mean age (± SD) | donors | Females/ males | total eyes | $CFB^{+}POS$ |
|---|---|---|---|---|---|
| AMD | 76.0 (±14.9) | 7 | 7/0 | 9 | 9/9 |
| Control | 77.0 (±11.7) | 3 | 1/2 | 4 | 0/4 |
